# Posttransplant complications: molecular mechanisms and therapeutic interventions

**DOI:** 10.1002/mco2.669

**Published:** 2024-09-02

**Authors:** Xiaoyou Liu, Junyi Shen, Hongyan Yan, Jianmin Hu, Guorong Liao, Ding Liu, Song Zhou, Jie Zhang, Jun Liao, Zefeng Guo, Yuzhu Li, Siqiang Yang, Shichao Li, Hua Chen, Ying Guo, Min Li, Lipei Fan, Liuyang Li, Peng Luo, Ming Zhao, Yongguang Liu

**Affiliations:** ^1^ Department of Organ transplantation The First Affiliated Hospital, Guangzhou Medical University Guangzhou China; ^2^ Department of Oncology Zhujiang Hospital Southern Medical University Guangzhou China; ^3^ Department of Organ transplantation Zhujiang Hospital Southern Medical University Guangzhou China

**Keywords:** infection, malignancy, organ transplantation, posttransplant complications, rejection, T cell

## Abstract

Posttransplantation complications pose a major challenge to the long‐term survival and quality of life of organ transplant recipients. These complications encompass immune‐mediated complications, infectious complications, metabolic complications, and malignancies, with each type influenced by various risk factors and pathological mechanisms. The molecular mechanisms underlying posttransplantation complications involve a complex interplay of immunological, metabolic, and oncogenic processes, including innate and adaptive immune activation, immunosuppressant side effects, and viral reactivation. Here, we provide a comprehensive overview of the clinical features, risk factors, and molecular mechanisms of major posttransplantation complications. We systematically summarize the current understanding of the immunological basis of allograft rejection and graft‐versus‐host disease, the metabolic dysregulation associated with immunosuppressive agents, and the role of oncogenic viruses in posttransplantation malignancies. Furthermore, we discuss potential prevention and intervention strategies based on these mechanistic insights, highlighting the importance of optimizing immunosuppressive regimens, enhancing infection prophylaxis, and implementing targeted therapies. We also emphasize the need for future research to develop individualized complication control strategies under the guidance of precision medicine, ultimately improving the prognosis and quality of life of transplant recipients.

## Graphical abstract

The most common posttransplant complications (PTC) include immune‐mediated complications (rejection and graft‐versus‐host disease), infections, metabolic complications (diabetes, hypertension, and dyslipidemia), and malignancies.

The mechanisms underlying PTC mainly involve allogeneic antigen‐activated innate immunity, cellular immunity, and humoral immunity responses, as well as the side effects and toxicity of immunosuppressants.

Treatment strategies for PTC include the use of immunosuppressants with fewer side effects, Treg cell therapy, infection screening and vaccination, mTOR inhibitors for cancer treatment, and CAR‐T cell therapy and Treg depletion therapy.

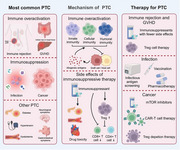

## 1 INTRODUCTION

Organ transplantation is the definitive therapeutic option for chronic and end‐stage organ failure, which can significantly prolong patient survival.^1^, ^2^ However, the improvement in long‐term survival of transplant recipients is limited, primarily attributed to the negative impact of posttransplant complications.^3^, ^4^ Although modern immunosuppressive regimens have been effective in reducing the incidence of acute rejection, immune‐mediated complications, such as chronic rejection and chronic graft insufficiency, remain a major challenge in organ transplantation.^5^ Moreover, infectious complications pose another major threat to the lives of transplant recipients. Immunosuppressive therapy, while reducing the risk of rejection, simultaneously increases the incidence of infection.^6^ Metabolic complications, such as posttransplant diabetes, dyslipidemia, and hypertension, also significantly impact the prognosis of recipients.^7^, ^8^, ^9^ The increasing incidence of posttransplant malignancies, concomitant with the prolonged survival of transplant recipients, is of particular concern.^10^ In view of these challenges, posttransplantation complications have become a major hurdle, limiting the further development of transplantation medicine and hindering the long‐term benefits for transplant recipients.

In‐depth investigation into the mechanisms underlying posttransplantation complications is of paramount importance for guiding clinical practice and improving transplantation outcomes. In recent years, advancements in molecular biology techniques have greatly facilitated the study of molecular mechanisms associated with posttransplantation complications. Regarding immune‐mediated complications, the roles of immune response pathways involving T cells and B cells, as well as cytokine and chemokine networks, in graft rejection and injury have been gradually elucidated.^5^, ^11^, ^12^ Studies on immunosuppression have further elucidated the mechanisms by which immunosuppressive agents induce metabolic disorders and organ toxicity, as well as the immunological basis for their propensity to promote infection and malignancy.^1^, ^13^, ^14^ These research advances have yielded novel insights and strategies for preventing and treating posttransplantation complications. However, our current understanding of posttransplantation complications remains limited and lacks depth, with many aspects requiring further investigation.

This review aims to provide a comprehensive summary of the clinical features and risk factors of posttransplantation complications, with a focus on the molecular mechanisms underlying their development, and to explore potential prevention and intervention strategies based on these findings. The paper is divided into three parts: (1) a summary of the epidemiological characteristics, clinical manifestations, and risk factors of major posttransplant complications, including immune‐mediated complications, infectious complications, metabolic complications, and malignant cancers; (2) an in‐depth examination of the molecular mechanisms underlying posttransplant complications, encompassing immune dysregulation, immunosuppressant side‐effects, and the molecular pathways of graft injury; and (3) a discussion of potential prevention and intervention strategies. Building upon the foundation laid by the first two parts, the third part summarizes the research progress in the prevention and treatment of posttransplantation complications and provides an outlook on future research directions.

## 2 MAJOR POSTTRANSPLANT COMPLICATIONS

Posttransplantation complications encompass a wide range of conditions that significantly impact the long‐term survival and quality of life of transplant recipients. This section provides a comprehensive overview of the major posttransplant complications, including immune‐mediated complications, infectious complications, metabolic and cardiovascular complications, malignancies, and other notable complications. By discussing the epidemiology, clinical manifestations, and risk factors associated with each complication category, this section lays the foundation for understanding the complex pathophysiology and management challenges in the posttransplantation setting.

### 2.1 Immune‐mediated complications

Posttransplant immune‐mediated complications, primarily consisting of rejection and graft‐versus‐host disease (GVHD), pose a significant challenge in the field of organ transplantation. Despite substantial progress in immunosuppressive therapy, rejection and GVHD continue to be critical factors impacting long‐term graft and patient survival.

Rejection can occur in nearly all types of allogeneic organ transplantation. Based on the onset timing and underlying mechanisms, rejection can be classified as hyperacute, acute, or chronic.^15^ Hyperacute rejection typically manifests within minutes to hours posttransplantation and is primarily mediated by preexisting donor‐specific antibodies, such as those encountered in ABO blood group‐incompatible kidney transplantation.^16^ Acute rejection most commonly develops days to weeks posttransplantation and is characterized by a cytotoxic response driven by donor‐specific lymphocytes, accompanied by humoral immune responses. Although the incidence of acute rejection following kidney and other organ transplantation has been markedly reduced with the use of immunosuppressive agents, the resulting graft damage and delayed graft function (DGF) continue to jeopardize recipient outcomes.^17^ Chronic rejection, occurring months to years posttransplantation, is a progressive pathological process characterized by graft vasculopathy and interstitial fibrosis. In solid organ transplantation (SOT), such as heart and other organs, these pathological changes can manifest early and exhibit a high incidence in the late posttransplant period. For instance, within 1 year following kidney transplantation, over 81% of patients develop interstitial fibrosis and tubular atrophy lesions stemming from cellular arteritis, with these lesions progressing to severe damage in more than 50% of transplanted kidneys within 5 years.^18^

GVHD is one of the most prevalent and severe complications following allogeneic hematopoietic stem cell transplantation (allo‐HSCT), frequently occurring in cases of incomplete donor–recipient (D–R) human leukocyte antigen (HLA) matching. Consequent to this histocompatibility disparity, immunocompetent cells within the graft mount an attack against recipient tissues, leading to dysregulation and dysfunction.^19^ Based on the onset timing and clinical manifestations, GVHD can be classified into two forms: acute and chronic. Acute GVHD typically manifests within 100 days posttransplantation, primarily affecting the skin, liver, and gastrointestinal tract. In contrast, chronic GVHD usually develops beyond 100 days posttransplantation and can involve any organ system.^20^ Research indicates that the incidence of acute GVHD ranges from 28 to 40% in moderate‐to‐severe cases, while chronic GVHD can affect up to 70% of patients.^21^, ^22^ As one of the primary factors influencing allo‐HSCT outcomes, GVHD is associated with a mortality rate of up to 25% in patients with chronic GVHD.^23^ Despite the implementation of various preventive and therapeutic approaches, including immunosuppressive agents and cellular therapies, GVHD continues to pose a significant challenge in the context of allo‐HSCT.

### 2.2 Infectious complication

Posttransplant infections contribute significantly to morbidity and mortality among transplant recipients. Approximately 70% of kidney transplant recipients are estimated to develop an infection within 3 years posttransplantation.^24^ Following cardiovascular disease, infections are the second most common cause of mortality in transplant recipients. In the late posttransplant period (4–10 years), infections are the primary cause of mortality, particularly among diabetic patients.^25^ Immunosuppressive therapy, administered to prevent rejection, increases the risk of infections in transplant recipients, primarily including donor‐derived infections, nosocomial and community‐acquired infections, and reactivation of latent pathogens.^26^ Frequently encountered bacterial infections comprise those caused by gram‐negative bacteria, such as Escherichia coli and Klebsiella pneumoniae, and gram‐positive bacteria, including staphylococci and enterococci. These infections can affect various organ systems and are strongly associated with the type of transplanted organ. For instance, urinary tract infections are linked to kidney transplantation, intra‐abdominal infections to liver transplantation, and pneumonia to lung transplantation.^27^ Cytomegalovirus (CMV) is the most prevalent viral infection following transplantation, potentially leading to fever, leukopenia, and organ involvement.^28^ Epstein–Barr virus (EBV), herpes simplex virus (HSV), varicella‐zoster virus (VZV), human herpesviruses (HHVs), and other human viruses are frequently encountered viruses in the posttransplantation period. In addition to the aforementioned viruses, human herpesvirus (HHV)‐6 and BK virus can also cause infections in transplant recipients.^29^ In general, Pneumocystis jirovecii pneumonia and candidiasis are prevalent fungal infections following transplantation, whereas SOT recipients infected with invasive fungi, such as Aspergillus and Mucorales species, experience higher morbidity and mortality rates.^30^, ^31^ The incidence of posttransplant infections exhibits a distinct temporal pattern. In the first month posttransplantation, infections are commonly associated with surgical complications, nosocomial exposures, and donor‐derived pathogens, including multidrug‐resistant bacteria such as methicillin‐resistant Staphylococcus aureus, vancomycin‐resistant enterococci, carbapenem‐resistant Enterobacteriaceae, and Clostridioides difficile. Opportunistic infections are more prevalent between 1 and 6 months posttransplantation, coinciding with the period of more intensive immunosuppression. During this time, reactivation of latent pathogens, such as BK virus, hepatitis C virus (HCV), and Mycobacterium tuberculosis, may also occur. Pneumocystis jirovecii pneumonia, herpesvirus infections (including CMV, HSV, VZV, and EBV), and hepatitis B virus (HBV) infections are less common during this period due to the use of prophylactic medications. Beyond 6 months posttransplantation, the majority of patients are on lower levels of immunosuppression, and infections are primarily community acquired. Moreover, there remains a risk of recurrent infections and delayed‐onset viral infections, particularly CMV.^32^, ^33^ Consequently, pretransplantation screening of donors and recipients, immunization, optimal antimicrobial and antiviral prophylaxis, and prudent use of antibiotics can mitigate the impact of infections. Furthermore, screening donors for bacterial infections, such as urinary tract infections and bacteremia, is essential.

### 2.3 Metabolic and cardiovascular complications

Posttransplant diabetes mellitus (PTDM) is a prevalent endocrine metabolic disorder following SOT in adults, affecting 10% to 40% of recipients. New‐onset diabetes after transplantation (NODAT), defined as diabetes that develops posttransplantation in patients without a prior history of diabetes, is a common and severe complication following transplantation of the kidney, liver, heart, and various other organs.^34^ NODAT‐related complications develop more rapidly compared with those in the general population with type 2 diabetes. Moreover, NODAT is associated with poorer outcomes, including an elevated risk of major cardiovascular events, graft failure, and mortality.^35^ The primary pathogenic mechanism underlying NODAT is believed to be β‐cell dysfunction. Other pathophysiologic processes involved in NODAT development include impaired insulin sensitivity and uncontrolled glucagon release.^36^, ^37^, ^38^, ^39^ Notable risk factors for NODAT include advanced age and obesity.^40^, ^41^ Importantly, the use of posttransplant immunosuppressive agents, such as glucocorticoids, calcineurin inhibitors (CNIs), and mammalian target of rapamycin (mTOR) inhibitors, is strongly associated with the development of NODAT.^42^, ^43^

Dyslipidemia and hypertension are prevalent metabolic complications among various solid organ transplant recipients. Dyslipidemia, encompassing both hyperlipidemia and hypercholesterolemia, exhibits a high incidence following renal, hepatic, and cardiac transplantation.^44^, ^45^, ^46^ In addition to nonmodifiable risk factors, such as advanced age and genetic predisposition, the use of immunosuppressive agents, including corticosteroids, CNIs, and mTOR inhibitors, are significant potential contributors to posttransplant dyslipidemia.^47^ Hypertension is one of the most frequent cardiovascular complications following renal, cardiac, and pulmonary SOT.^48^, ^49^, ^50^ The presence of common risk factors for hypertension in recipients, such as alcohol abuse, smoking, and obesity, along with transplant renal artery stenosis, allograft rejection, and immunosuppression, are contributing factors to posttransplant hypertension.^51^, ^52^, ^53^ Notably, considering the substantial cardiovascular damage caused by dyslipidemia and hypertension, the management of blood lipids and blood pressure is crucial, particularly in heart transplant recipients.^54^, ^55^

Cardiovascular disease is the primary cause of mortality in early SOT recipients.^56^ The risk of cardiovascular complications, such as coronary artery disease, heart failure, arrhythmias, and pulmonary hypertension, is substantially higher in the transplant population compared with the general population.^51^ Moreover, factors such as smoking and obesity are also prevalent risk factors for posttransplant cardiovascular disease.^57^ Notably, the aforementioned conditions, including diabetes, hyperlipidemia, hypertension, and the closely associated use of immunosuppressive agents, are all regarded as risk factors for the development of posttransplant cardiovascular disease.^58^

### 2.4 Malignant cancer

Posttransplantation malignancies are among the most common postoperative complications in SOT and HSCT recipients, with an overall prevalence ranging from 4 to 18%.^1^ High‐risk types of de ^novo posttransplant malignancies^ include skin cancer, genitourinary cancers, and posttransplant lymphoproliferative disease (PTLD).^59^ In addition to traditional risk factors, such as smoking, sun exposure, and family history of cancer, the transplant population has unique risk factors for malignancy, including immunosuppression, oncogenic viruses, and donor‐derived transmission.^60^ Epidemiologic data demonstrate that the incidence of de novo malignancies within 10 years posttransplantation is two to three times higher in transplant recipients compared with the general population, with the incidence of nonmelanoma skin cancers being up to 13 times higher than in the nontransplant population.^61^ In general, the prognosis of de novo malignancies following transplantation is poor, with the 5‐year survival rate being substantially lower compared with patients with similar malignancies in the general population. At present, malignancies have become a leading cause of long‐term mortality in transplant recipients, necessitating close follow‐up, and surveillance.

In addition to novo neoplastic malignancies, virus‐associated cancers constitute a significant category of posttransplantation malignancies. Frequent viral infection‐associated posttransplantation malignancies include EBV‐associated PTLD, HHV‐8‐associated Kaposi's sarcoma (KS), and human papillomavirus‐associated skin cancer, anogenital cancer, and head and neck cancer.^62^, ^63^, ^64^ Among these, PTLD, one of the most common posttransplantation malignancies, is associated with EBV infection in approximately 55−65% of cases.^65^, ^66^ Upon entering the human body, EBV typically first infects the oropharyngeal epithelial cells. As the viral infection subsides, the small amount of EBV present in B cells enters a latent period, evading the host immune system. Following reactivation of the virus due to various factors, the lifespan of the virus‐infected B lymphocytes is prolonged, with an increased potential for mutations.^65^, ^67^, ^68^ Patients with early‐onset PTLD are typically EBV‐positive and have a remarkably high mortality rate.^69^, ^70^ Moreover, HHV‐8‐associated virologic markers are positive in nearly all transplant recipients with KS.^64^ KS in the immunosuppressed state is typically more aggressive and fatal compared with immunocompetent recipients.^71^ Notably, all malignancies with a substantially increased risk compared with the general population were associated with viruses, underscoring the crucial role of oncogenic viruses in posttransplant cancers.^60^

### 2.5 Other complications

DGF is one of the most common early complications following kidney transplantation, with an incidence of approximately 25−30%.^72^ Although there are various definitions of DGF, the most widely accepted one is the failure of the transplanted kidney to function at the expected level within 7 days posttransplantation, often necessitating dialysis.^73^ The occurrence of DGF is closely associated with donor factors (e.g., advanced age, deceased donor transplantation), recipient factors (e.g., immune hyperreactivity, recurrence of kidney disease), and perioperative factors (e.g., ischemia–reperfusion injury, immunosuppressant toxicity).^74^ Notably, both acute and chronic rejection are important causes of DGF exacerbation, which significantly reduces posttransplant survival.^75^ Substantial clinical evidence demonstrates a strong association between DGF and subsequent chronic allograft dysfunction.^76^, ^77^

Transplant recipients frequently experience a range of comorbid gastrointestinal and neurologic complications. Gastrointestinal symptoms, including nausea, vomiting, and diarrhea, may be associated with factors such as immunosuppressant medications (e.g., mycophenolate mofetil [MMF]) and infections (e.g., CMV enteritis).^78^, ^79^ Moreover, transplant recipients have an increased risk of developing severe gastrointestinal complications, such as diverticulitis and gastrointestinal perforation.^80^, ^81^ Targeted modification of immunosuppressive regimens, prevention and treatment of related infections, and symptomatic supportive care are essential for managing posttransplant gastrointestinal complications.^82^ Neurologic complications, such as headache, tremor, and sensory abnormalities, may be associated with immunosuppressant neurotoxicity (e.g., tacrolimus), infections (e.g., cryptococcal meningitis), and metabolic disturbances (e.g., hypomagnesemia).^83^, ^84^

## 3 MOLECULAR MECHANISMS OF POSTTRANSPLANTATION COMPLICATIONS

The molecular mechanisms underlying posttransplantation complications involve a complex interplay of immunological, metabolic, and oncogenic processes. This section delves into the intricate molecular pathways and cellular interactions that contribute to the development of immune‐mediated complications, the effects of immune oversuppression, and the pathogenesis of posttransplant malignancies. Additionally, it addresses posttransplant metabolic and cardiovascular complications. By providing a comprehensive analysis of the key molecular players and signaling cascades involved in each complication category, this section offers valuable insights into potential therapeutic targets and strategies for mitigating posttransplantation morbidity and mortality.

### 3.1 Posttransplant complications due to overimmunosuppression

**FIGURE 1:**
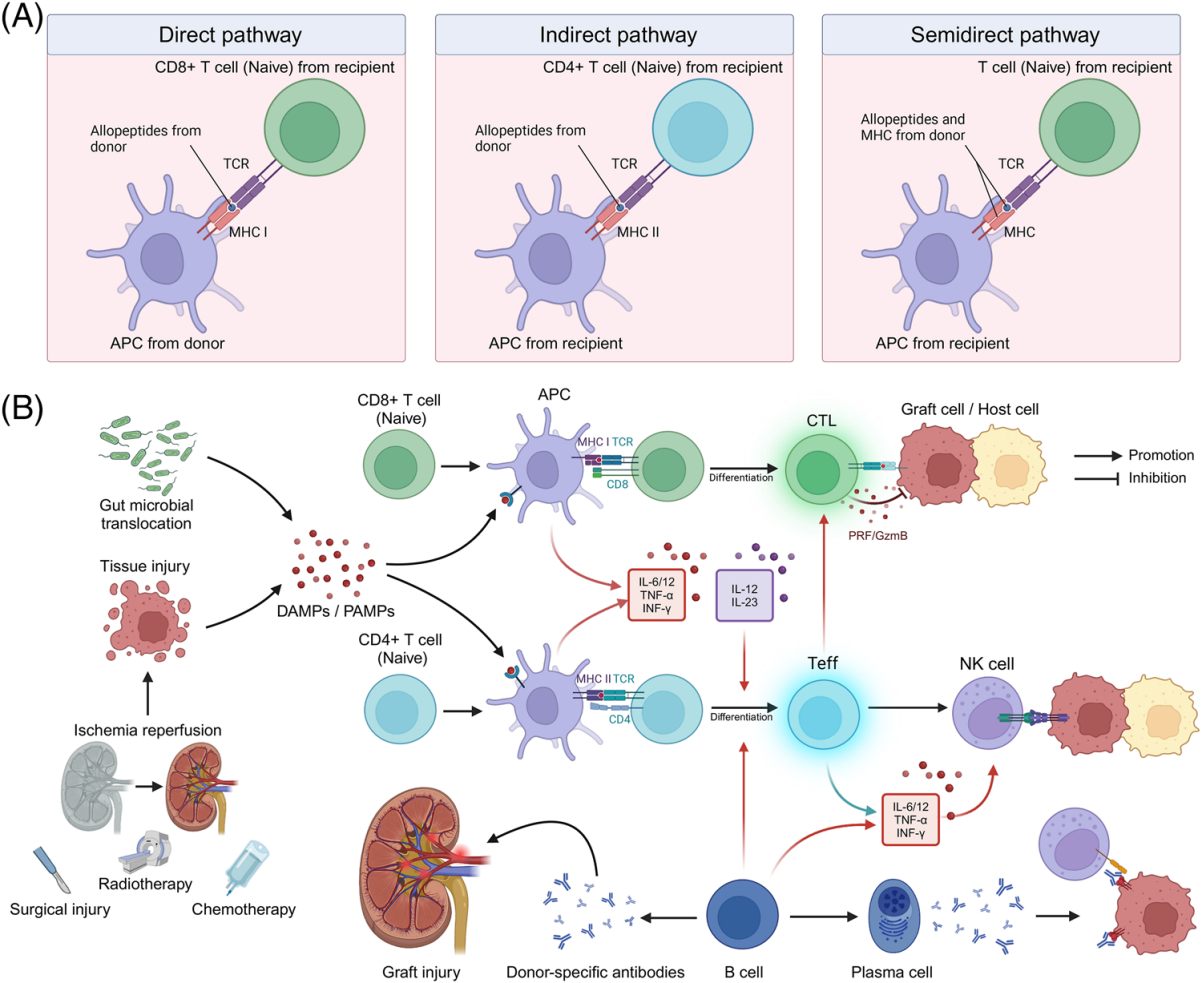
Mechanisms of complications associated with immune overactivation following transplantation. (A) Three pathways of allorecognition. In the direct pathway, donor antigen‐presenting cells (APCs) interact directly with recipient T cells. In the indirect pathway, recipient APCs present processed donor alloantigen peptides to recipient T cells, resembling a typical immune response. In the semi‐direct pathway, recipient APCs acquire donor HLA molecules that directly present peptides to recipient T cells. (B) Mechanisms of immune activation associated with transplantation. Tissue damage and the release of damage‐associated molecular patterns (DAMPs), such as ATP, uric acid, IL‐33, and HMGB‐1, can result from ischemia–reperfusion injury and surgical trauma during allogeneic solid organ transplantation, as well as preoperative chemotherapy and radiotherapy for hematopoietic stem cell transplantation. Intestinal barrier damage can also lead to alterations in gut microbiota and the release of pathogen‐associated molecular patterns (PAMPs). These DAMPs and PAMPs serve as danger signals that interact with the recipient's innate immune cell surface and intracellular pattern recognition receptors (PRRs), initiating and sustaining innate immune responses. Upon APC stimulation, naïve CD8+ T cells differentiate into cytotoxic T cells, which can destroy donor organs or host cells through the release of granules and perforin. Following APC stimulation, naïve CD4+ T cells differentiate into effector T (Teff) cells. Teff cells enhance the cytotoxic activity of natural killer (NK) cells and cytotoxic T lymphocytes (CTLs) by secreting proinflammatory cytokines, including IL‐6, IL‐12, and TNF‐α. B cells can function as specific antigen‐presenting cells and modulate CD4+ T cell activation via indirect allorecognition pathways. Furthermore, activated B cells can differentiate into plasma cells that produce antibodies, potentially leading to antibody‐mediated rejection (ABMR) through mechanisms such as antibody‐dependent cell‐mediated cytotoxicity (ADCC) and direct cytotoxic effects. Substantial quantities of donor‐specific antibodies produced by recipient B cells can bind to the vascular endothelium of the graft, rapidly inducing inflammatory infiltration and vascular damage. This figure was created using tools provided by Biorender.com (accessed July 2, 2024).

SOT and allo‐HSCT are crucial clinical treatments for numerous end‐stage diseases. However, the posttransplantation period is frequently characterized by severe immune complications, primarily manifested as allograft rejection and GVHD. Although the contexts of occurrence and clinical manifestations of these two complications differ, the underlying immune activation and effector mechanisms share substantial similarities. This section will systematically summarize and discuss the mechanisms underlying these two types of complications (Figure 1).

#### 3.1.1 Innate immune activation

Innate immunity plays a crucial role in the immune response to transplantation. In the context of SOT, ischemia–reperfusion injury, surgical trauma, and other factors result in extensive cell death within the transplanted organ, leading to the release of damage‐associated molecular patterns (DAMPs), including high mobility group protein B1 (HMGB1), heat shock proteins, ATP, and extracellular DNA.^85^ Likewise, extensive tissue damage caused by pretransplant conditioning regimens for allo‐HSCT, such as radiotherapy and total body irradiation, can result in a substantial release of DAMPs, including ATP, uric acid, interleukin (IL)−33, and HMGB‐1.^86^ Moreover, damage to the intestinal barrier can lead to alterations in the gut microbiome, resulting in the translocation of gut microbes and the release of pathogen‐associated molecular patterns (PAMPs).^87^ These DAMPs and PAMPs serve as danger signals that can bind to pattern recognition receptors (PRRs) on the surface and within the cytoplasm of the recipient's innate immune cells, such as Toll‐like receptors and NOD‐like receptors, initiating and sustaining the innate immune response.^88^ Activation of PRRs triggers the activation of downstream inflammatory signaling pathways, including nuclear factor‐κB (NF‐κB) and other related pathways, and promotes the secretion of proinflammatory cytokines, such as IL‐1, IL‐6, and tumor necrosis factor‐alpha (TNF‐α).^89^, ^90^ In the context of graft rejection, these proinflammatory responses can lead to graft damage, resulting in acute or chronic rejection. Furthermore, activation of the complement system (e.g., C3a and C5a) can directly activate T cells and antigen‐presenting cells (APCs) within the graft.^91^ In the context of GVHD, innate immune activation promotes the production of stimulatory cytokines, such as IL‐12 and IL‐23, which facilitate the differentiation of donor T cells into various effector CD4+ T cell (Teff) lineages. Notably, in addition to their antigen‐presenting functions, neutrophils, as part of the innate immune system, are believed to directly damage gastrointestinal tissues through the release of reactive oxygen species.^92^

#### 3.1.2 Allogeneic antigen recognition

In the context of allogeneic rejection, the graft expresses donor‐derived alloantigens, primarily major histocompatibility complex (MHC) molecules and minor histocompatibility antigens.^93^, ^94^ The recipient's immune system recognizes these alloantigens through direct and indirect pathways, which subsequently initiate an alloimmune response.^95^ In the direct pathway, immature dendritic cells (DCs), which are prevalent in the donor organ, are stimulated by inflammatory signals to mature and migrate from the graft to the paracortex of the recipient's lymph nodes. These mature DCs exhibit high expression of MHC class I molecules and elevated levels of MHC costimulatory molecules, which can stimulate graft‐specific CD8+ T‐cell activation within the lymph nodes, thereby inducing an acute allogeneic rejection response. As donor APCs become depleted, the role of recipient APCs in the rejection response gradually becomes predominant. Graft antigens are recognized and processed by recipient APCs and subsequently bind to recipient MHC class II molecules. The antigenic peptide–MHC class II complex is then presented to CD4+ T cells, thereby inducing a slower and less intense immune response through this indirect pathway.^96^ Notably, a semi‐direct pathway also contributes to allogeneic rejection. In this pathway, recipient APCs directly acquire intact donor MHC molecules through cell‐to‐cell interactions and present them to recipient T cells. This pathway is characterized by recipient APCs presenting both donor and recipient MHC molecules, thereby activating T cells through both direct and indirect pathways, and plays a crucial role in both acute and chronic rejection.^97^, ^98^

During the development of acute GVHD, donor T cells, mediated by L‐selectin, CCR7, and other molecules, migrate to lymphoid organs or host tissues, where they proliferate and differentiate extensively upon stimulation by host antigens presented by APCs via MHC class I or class II molecules.^99^ Furthermore, costimulatory pathways, including CD28, CD278, and TNFR superfamily receptors (e.g., CD40L and OX40), are also essential for T cell activation.^100^, ^101^, ^102^, ^103^ Various cytokines, such as interferon‐gamma (IFN‐γ), TNF‐α, and others, play crucial roles in T cell activation. Among these cytokines, IFN‐γ promotes antigen presentation by upregulating the expression of adhesion molecules, chemokines, and HLA molecules. Moreover, inflammatory chemokines, such as C‐X‐C motif chemokine ligand (CXCL9), CXCL10, and CXCL11, can recruit activated Teff to infiltrate GVHD target organs.^104^

#### 3.1.3 T‐ and B‐cell‐mediated adaptive immune responses

T cells activated by allogeneic recognition or innate immune activation play a pivotal role in mediating the allogeneic immune response and inducing graft or host injury. The damaging effects of T cells on the graft or host primarily involve direct lysis of graft cells by cytotoxic T cells (CD8+ T cells) and the secretion of cytokines by helper T cells (CD4+ T cells) to promote inflammatory responses. Upon APC presentation and stimulation, which relies on T cell receptor (TCR) recognition of alloantigens, naïve CD8+ T cells are activated and rapidly proliferate and differentiate into effector T cells and memory T cells.^105^ Influenced by a specific cytokine microenvironment, the initial CD8+ T cells differentiate into various CD8+ T cell lineages, including IFN‐γ‐producing Tc1, IL‐4‐producing Tc2, IL‐9‐producing Tc9, IL‐17‐producing Tc17, and IL‐22‐producing Tc22 cell subpopulations.^106^, ^107^ Among these subpopulations, typical cytotoxic T cells (CTLs), such as Tc1, Tc2, and Tc22, can mediate rejection by expressing granzymes, perforin, and other cytotoxic molecules.^108^, ^109^, ^110^ In GVHD, CTLs are the primary effector cells that also mediate host cell lysis through the Fas/FasL pathway and cytotoxic molecules (e.g., perforin and granzyme B).^111^ In the indirect pathway of rejection and GVHD, MHC class II complexes presented by APCs in the draining lymph nodes stimulate naïve CD4+ T cells by binding to the TCR, inducing their proliferation and differentiation into various subpopulations of CD4+ helper T (Th) cells. The Th subpopulations exhibit varying levels of surface marker expression and produce different cytokines, which play a broad and complex role in the allogeneic rejection response and GVHD. Among these subpopulations, the subpopulation that recognizes and removes antigens while promoting the immune response is referred to as Teff.^112^ Common Teff include Th1 cells (which primarily secrete IFN‐γ, IL‐2, and TNF‐α, and mediate cellular immune responses), Th2 cells (which mainly secrete IL‐4, IL‐5, and IL‐13, promote B‐cell differentiation, maturation, and antibody production, and mediate humoral immune responses), Th17 cells (which primarily secrete IL‐17 and IL‐22), and T follicular helper cells (which produce IL‐21 and promote humoral immunity in germinal centers).^113^, ^114^, ^115^

In immune rejection, memory T cells in transplant recipients can be derived from alloreactive memory T cells generated by previous exposure to alloantigens (e.g., blood transfusion, pregnancy, or a previous transplant) or from the differentiation of CD8+ T cells activated by direct or indirect pathways after transplantation.^116^ Notably, viral or other microbial infections can also induce the generation of alloreactive memory T cells through antigen‐mimetic mechanisms, enabling these cells to cross‐recognize allogeneic antigens and participate in transplant rejection.^117^ Compared with naïve T cells, these memory T cells have a lower activation threshold, a more rapid initiation effect, and enhanced proliferation, differentiation, and cytokine secretion.^118^, ^119^ These properties enable memory T cells to play a more prominent role in transplant rejection.

B cells also play a crucial role in allogeneic rejection immunization in transplantation. On the one hand, B cells can function as specific APCs and influence the activation of CD4+ T cells through the indirect recognition pathway.^120^, ^121^ For instance, heart transplantation in a mouse model with defective indirect presentation of alloantigens by B cells resulted in significantly prolonged survival of heart grafts, highlighting the importance of B cell presentation in mediating graft rejection.^122^ Moreover, B cells play a vital role in the proliferation and subsequent differentiation of alloreactive T cells into memory T cells, thereby accelerating subsequent rejection.^123^ In this process, a wide array of cytokines (e.g., IL‐6 and IFN‐γ) secreted by B cells play a crucial stimulatory role.^124^ On the other hand, activated B cells can differentiate into plasma cells that produce antibodies, potentially leading to antibody‐mediated rejection. These antibodies can damage the graft through various mechanisms, such as complement‐dependent cytotoxicity, antibody‐dependent cell‐mediated cytotoxicity (ADCC), and direct cytotoxic effects. In hyperacute rejection, preexisting donor‐specific antibodies in the recipient's blood bind to the endothelium of the graft vasculature and activate the complement system. This initiates a cascade of pathological processes, including neutrophil infiltration, vascular injury, hemorrhage, fibrin deposition, and platelet aggregation, ultimately leading to irreversible graft damage within a short period.^125^, ^126^ In ADCC, the Fab segment of the alloantibody binds to the antigen on the graft cell surface, while its Fc segment cross‐links with the Fc receptor on the surface of the recipient's effector cells (e.g., natural killer [NK] cells and macrophages), activating them and inducing the release of cytotoxic substances, ultimately damaging the graft.^127^, ^128^ Furthermore, alloantibodies can directly induce apoptosis or necrosis by binding to specific antigens on the graft cell surface.^129^

#### 3.1.4 Disorders of immune regulation

Regulatory T cells (Tregs) are a subpopulation of T cells that employ various inhibitory mechanisms to regulate the activity of other immune cells and modulate immune system homeostasis.^130^ Research has demonstrated that one of the primary functions of Tregs is to suppress the activation, proliferation, and cytokine production of effector T cells.^131^, ^132^ By inducing and maintaining transplantation tolerance, Tregs play a crucial role in preventing or attenuating immune rejection and GVHD. Consequently, when the number or function of Tregs is reduced or suppressed, it may disrupt immune homeostasis, leading to rejection and GVHD. Numerous observational studies in various transplant types, such as kidney and liver, have suggested a correlation between the relative reduction of Tregs and short‐term rejection after transplantation.^133^, ^134^ Likewise, several studies have observed an insufficient upregulation of Tregs in target organ biopsies of GVHD patients, such as the intestines and skin.^135^, ^136^ At present, numerous preclinical animal experiments and clinical trials have demonstrated that Tregs play a pivotal role in preventing severe GVHD and rejection.^137^, ^138^, ^139^

### 3.2 Posttransplant complications due to under‐immunosuppression

**FIGURE 2:**
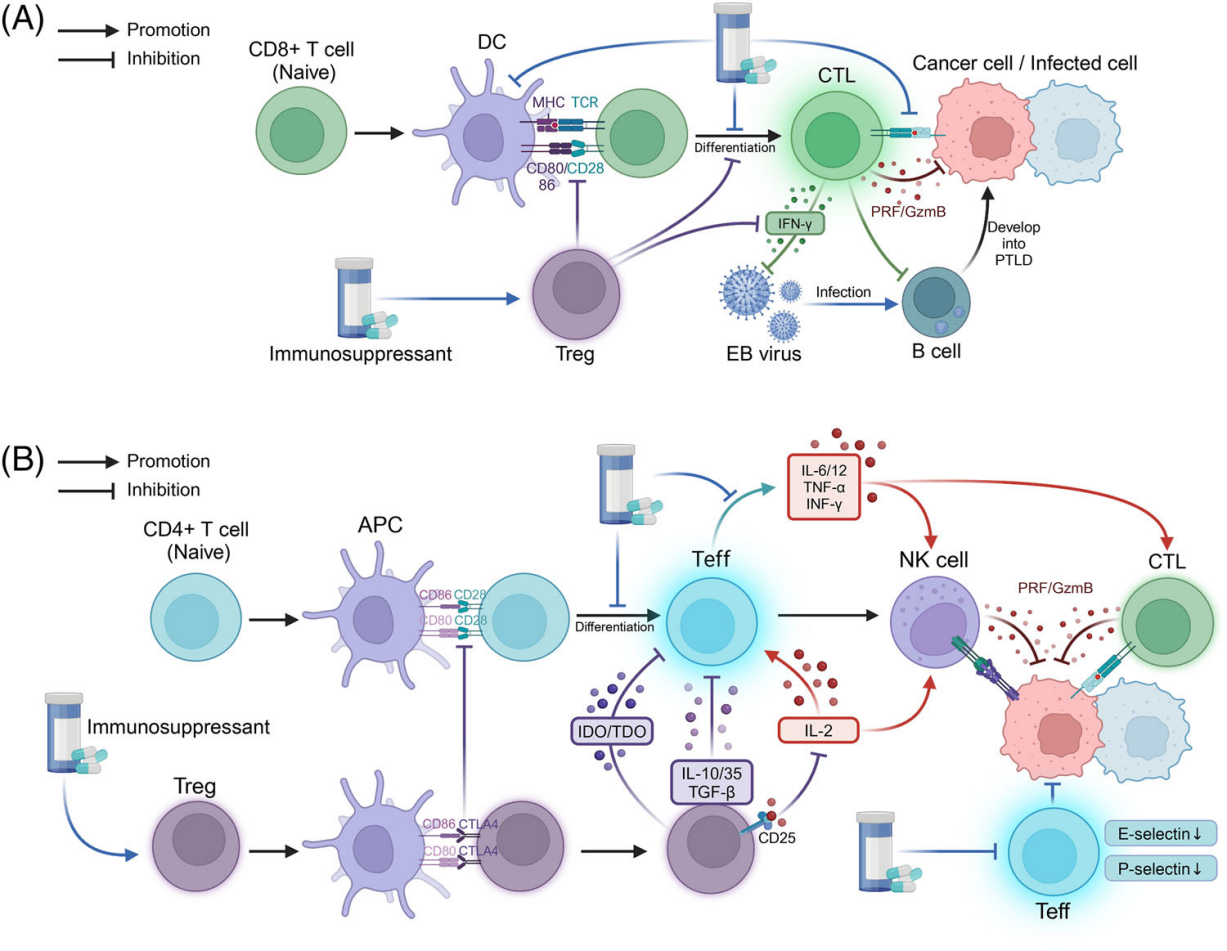
Mechanisms of complications associated with immunosuppression following transplantation. (A) Under normal circumstances, antigen‐presenting cells (APCs) stimulate the differentiation of naïve CD8+ T cells into cytotoxic T cells, which can eliminate infected or malignant cells through the release of granules and perforin. However, the excessive use of immunosuppressive agents can disrupt this process. Immunosuppressants can inhibit the activation, differentiation, and cytotoxic function of naïve CD8+ T cells. Moreover, immunosuppressants can activate regulatory T cells (Tregs), which may hinder the development of naïve CD8+ T cells and the production of interferon‐γ (IFN‐γ) by cytotoxic T cells. Furthermore, reduced IFN‐γ secretion may elevate the risk of Epstein–Barr virus (EBV) infection in B cells, consequently promoting the development of EBV‐associated posttransplant lymphoproliferative disorder (PTLD). Additionally, the suppression of cytotoxic T cell function against EBV further increases the risk of posttransplant malignancies. (B) When the body's antitumor immune system is functioning optimally, APCs stimulate the differentiation of naïve CD4+ T cells into effector T (Teff) cells. Teff cells enhance the anti‐infective and antitumor effects of natural killer (NK) cells and cytotoxic T lymphocytes (CTLs) by secreting proinflammatory cytokines, including IL‐6, IL‐12, and TNF‐α. However, the overuse of immunosuppressive agents can disrupt this process by inhibiting the differentiation of naïve CD4+ T cells and impairing the ability of Teff cells to secrete proinflammatory cytokines. Moreover, excessive immunosuppression promotes the competitive binding of cytotoxic T‐lymphocyte‐associated protein 4 (CTLA‐4) on Teff cells to CD80/86 on APCs, thereby inhibiting the activation of naïve CD4+ T cells. Furthermore, Tregs suppress Teff cell function and subsequent antitumor effects by secreting immunomodulatory metabolites (e.g., indoleamine 2,3‐dioxygenase [IDO] and tryptophan 2,3‐dioxygenase [TDO]) and anti‐inflammatory cytokines (e.g., IL‐10 and IL‐35), as well as by inhibiting IL‐2 production. This figure was created using tools provided by Biorender.com (accessed July 2, 2024). GzmB, granzyme B; IDO, indoleamine 2,3‐dioxygenase; PRF, perforin; TDO, tryptophan 2,3‐dioxygenase.

Immunosuppressive therapies play a crucial role in transplantation antirejection therapy; however, they can also cause severe adverse reactions through various pathways. A comprehensive understanding of the mechanisms of action of immunosuppressive therapies is essential for optimizing immunosuppressive regimens and minimizing the risk of complications. This section will explore the molecular mechanisms underlying the heightened susceptibility to infections and increased risk of tumorigenesis associated with immunosuppressive therapies (Table 1 and Figure 2).

**TABLE 1:** Summary of mechanisms for side effects of immunosuppressive therapy.

Complications	Major mechanism	Details	References
PTDM	Immunosuppressive therapy‐related metabolic disorders	Increased insulin resistance occurs in peripheral tissues	172, 175
		Direct damage occurs to pancreatic β‐cells	173, 174
Dyslipidemia		Key enzymes are upregulated in the cholesterol biosynthesis process	177
		LDL receptor binding to LDL is interfered with, and lipoprotein lipase activity is reduced	178
Hypertension		Vasodilators are decreased, and vasoconstrictors are increased	180, 181
		Aldosterone levels are mediated, and sympathetic nerve activation leads to sodium retention	182, 183, 184
Infection and malignancy	Immunosuppressive therapy‐induced impaired immune surveillance	Proliferation and activation are inhibited in CD8+ T cells and CD4+ T cells	140, 141
		Th17 cell differentiation is inhibited	142, 143
		Adhesion and penetration capabilities are reduced in CD8+ T cells	144, 145, 146
		Proinflammatory cytokine release is reduced	147, 148
		Cellular communication is suppressed, impacting T cell immune effects	150
	Immunosuppressive therapy‐induced enhancement of Tregs immunosuppressive function	Immunosuppressants affect Foxp3 expression level	153, 154
		Co‐inhibitory receptors, such as TIGIT, PD‐1, CTLA‐4, and so on, are overexpressed by Tregs	155, 156, 157, 158
		Immunosuppressive cytokine secretion by Tregs occurs, inhibiting Teff cells and consuming IL‐2	159
		Cytotoxic substance and immunosuppressive metabolite production is increased in Tregs	160, 161

Abbreviations: CTLA‐4, cytotoxic T lymphocyte‐associated antigen‐4; IL‐2, interleukin‐2; LDL, low‐density lipoprotein; PD‐1, programmed cell death protein‐1; PTDM, posttransplantation diabetes mellitus; TIGIT, T cell immunoreceptor with immunoglobulin and ITIM domain.

#### 3.2.1 Impaired immune surveillance

Transplant recipients require long‐term use of immunosuppressive drugs to prevent rejection; however, this also leads to impaired immune surveillance function and an increased risk of infection and malignancy. Immunosuppressants inhibit the function of various types of immune cells, including T lymphocytes, through multiple mechanisms, thereby weakening the body's defense against pathogenic microorganisms and cancer cells.

Dysfunction of T cells, including CD8+ T cells and CD4+ T cells, is a crucial component of impaired transplant‐associated immune surveillance. The use of immunosuppressive agents can impair the proliferation and activation of CD8+ T cells and Teff, thereby diminishing their immune effects. Common immunosuppressive agents include CNIs, inosine monophosphate dehydrogenase inhibitors, and dihydroorotate dehydrogenase inhibitors, among others. For instance, even at very low doses, everolimus significantly inhibits the proliferation of CD8+ T cells.^140^ Cyclosporine has been demonstrated to inhibit the maturation and differentiation of thymocytes by suppressing protein kinase C activation and reducing CD4/CD8 expression levels.^141^ For Teff, the inhibition of Tim‐1 ligand, which is used to inhibit chronic rejection of cardiac grafts, is believed to be closely associated with the inhibition of Th17 cell differentiation and development.^142^ Likewise, 15‐deoxyspergualin effectively inhibits the growth of naïve CD4+ T cells following early activation and reduces their polarization towards Th1 effector cells.^143^ Furthermore, immunosuppressive agents also impact lymphocyte‐mediated immunity. In the presence of IL‐2, cyclosporine inhibits the reactivation of quiescent, antigen‐dependent cytotoxic T cells and directly suppresses their effector phase.^144^ Likewise, when ex vivo expanded cytotoxic somatic cells were exposed to cyclosporine for one week, a significant decrease in their ability to lyse target cells was observed.^145^ Moreover, MMF inhibited the adhesion and penetration of CD8+ T cells to target effector cells, which may be related to the downregulation of specific endothelial membrane molecules and the loss of protein localization in lymphocyte protrusions.^146^ Similarly, the reduced release of proinflammatory cytokines due to immunosuppression decreases the immune function of Teff, including IFN‐γ, TNF‐α, IL‐12, and IL‐6.^147^, ^148^ Additionally, immunosuppressive agents may also inhibit the direct cytotoxic effects of Teff cells, such as adhesion and infiltration.^146^ Notably, the inhibition of cells that communicate with T cells may affect their immune effects.^149^ For instance, kinsenoside, a potential immunosuppressive drug for autoimmune hepatitis, can inhibit CD8+ T cell activity by reducing crosstalk between the metabolism‐associated PI3K–AKT pathway and the inflammation‐associated JAK2–STAT3 pathway in DCs.^149^

For transplant patients, the use of immunosuppressants such as mTOR inhibitors and prednisolone is the main cause of elevated levels of Tregs, which can inhibit the function of effector T cells, such as CD8+ T cells and Th cells, through multiple molecular pathways.^150^, ^151^ Forkhead box P3 (Foxp3), a transcription factor, is highly associated with Tregs and is responsible for regulating their development and function.^130^, ^131^, ^132^, ^133^, ^134^, ^135^, ^136^, ^137^, ^138^, ^139^, ^140^, ^141^, ^142^, ^143^, ^144^, ^145^, ^146^, ^147^, ^148^, ^149^, ^150^, ^151^, ^152^ Common posttransplantation immunosuppressants, such as cyclosporine A and rapamycin (RAPA), can affect the expression level of Foxp3, thereby modulating the function of Tregs. For instance, Qu et al.^153^ demonstrated that RAPA significantly increased the ratio of Foxp3+ Treg cells to CD4+ T cells in the spleen and thymus of mice. Battaglia et al.’s^154^ study showed that RAPA selectively expanded naturally occurring Foxp3+ Tregs in mice in vitro, and these Tregs could inhibit the proliferation of homologous T cells. Moreover, in the presence of immunosuppressive agents, Tregs can exert regulatory immune effects by overexpressing coinhibitory receptors, such as T‐cell immunoglobulin and ITIM domain (TIGIT), programmed cell death protein 1 (PD‐1), and CTL‐associated protein 4 (CTLA‐4).^155^, ^156^ For instance, Zeng et al.^157^ demonstrated that the immunosuppressive function of Tregs was more potent after the combination of tacrolimus and MMF compared with tacrolimus alone, and the immunosuppressive effect of Tregs was attenuated by the use of PD‐1 or TIGIT antibodies. In vitro cellular experiments by Chen et al.^158^ showed that the treatment of Tregs with RAPA can upregulate the expression of PD‐1‐related mRNA in mice. In the context of transplantation‐associated immunosuppression, the expanded Tregs can inhibit the proliferation and effects of effector T cells through the secretion of immunosuppressive cytokines, such as IL‐10, transforming growth factor‐β, and IL‐35. Moreover, the high expression of CD25 (IL‐2 receptor α chain) on Tregs enables them to deplete IL‐2 from the surrounding environment via their high‐affinity IL‐2 receptor, thereby limiting the binding of other effector T cells to IL‐2 and the subsequent activating effect.^159^ Furthermore, Tregs can produce cytotoxic substances that act on effector T cells, such as granzymes and perforin, which directly inhibit their number and function.^160^ Tregs‐associated immunosuppressive metabolites, such as indoleamine 2,3‐dioxygenase, and tryptophan 2,3‐dioxygenase, also inhibit the function and proliferation of effector T cells.^161^

In conclusion, immunosuppressive agents impair cellular immunity in transplant recipients through multiple mechanisms, leading to compromised immune surveillance. First, the functions of key effector cells, such as CD8+ T cells and effector CD4+ T cells, which are crucial for anti‐infection and anticancer responses, are attenuated. Second, the immunosuppressive effects of Tregs are relatively enhanced. This immunosuppression‐associated impairment of immune surveillance is a significant contributor to the increased risk of infection and malignancy in transplant recipients. An in‐depth investigation of the molecular mechanisms underlying immunosuppression and impaired immune surveillance in transplant recipients can facilitate the optimization of immunosuppressive regimens. The goal is to prevent rejection while preserving the body's defense mechanisms to the greatest extent possible, ultimately improving transplantation outcomes.

#### 3.2.2 Correlation between infection and carcinogenesis

The close association of CD8+ T cells with EBV infection‐associated PTLD has garnered attention, considering the crucial role of viral infection in posttransplantation tumorigenesis and progression. During EBV‐associated PTLD, the activation of EBV‐specific CD8+ T cells acts as a potent cancer suppressor, exerting cytotoxic effects early in the infection and maintaining EBV suppression over time.^162^ However, although EBV stimulation is usually accompanied by an increase in CD8+ T cell numbers and hyperfunction, CTLs can be infected by EBV during the killing of EBV‐infected target cells in the presence of extensive, uncontrolled EBV replication, which may impair the cytotoxic function of CTLs.^163^, ^164^ For instance, Tanaka et al.^165^ reported a case of a patient with severe aplastic anemia who had a persistently high peripheral blood EBV‐associated DNA load after allo‐HSCT; the patient was characterized by oligoclonal TCR Vβ profiles in peripheral lymphocytes after treatment with rituximab, suggesting that CTLs may be affected by EBV infection.^165^ Moreover, in immunocompetent hosts, the EBV genome is immortalized and forms episomes in latently infected B cells.^166^ When transplantation‐associated immunosuppression leads to a decline in the function of T cells, such as CTLs, the concomitant dysfunctional activation of antiviral functions, such as a decrease in IFN‐γ production, may be accompanied by uncontrolled proliferation of B cells, thereby promoting the development of B‐cell‐derived PTLD.^166^, ^167^

### 3.3 Metabolic and cardiovascular complication

Metabolic and cardiovascular complications are significant concerns in the posttransplant period, substantially impacting patient outcomes and quality of life. These complications arise from a complex interplay of factors, including the effects of immunosuppressive medications, infections, and the underlying health conditions of transplant recipients. Understanding the mechanisms behind these complications is crucial for developing effective prevention and treatment strategies. This section will explore two major contributors to posttransplant metabolic and cardiovascular complications: drug‐induced metabolic disorders and toxicity, and the critical role of infections.

#### 3.3.1 Drug‐induced metabolic disorders and toxicity

Immunosuppressive drugs play a crucial role in the prevention and treatment of GVHD; however, they can also cause metabolic disorders and toxic reactions through various mechanisms. Among the numerous immunosuppressive agents, a growing body of research has demonstrated that CNIs and mTOR inhibitors carry the potential risk of causing PTDM.^168^, ^169^, ^170^, ^171^ Numerous animal experiments and clinical studies suggest that the core pathogenesis of immunosuppressant‐associated PTDM involves immunosuppressants causing abnormal glucose tolerance through direct damage to pancreatic β‐cells and increased insulin resistance in peripheral tissues. For instance, a clinical study by Duijnhoven et al.^172^ demonstrated a significant decrease in insulin sensitivity and secretion in renal transplant patients receiving tacrolimus. Conversely, a study by Boots et al.^173^ found that steroid withdrawal reduced insulin resistance levels in renal transplant patients, and that reduced tacrolimus levels increased pancreatic β‐cell secretion. Furthermore, mouse experiments by Shivaswamy et al.^174^ demonstrated the damaging effects of tacrolimus treatment on pancreatic β‐cells and the inhibitory effects of sirolimus on insulin signaling. Likewise, Larsen et al.^175^ observed tacrolimus‐ and sirolimus‐induced insulin resistance in a rat model. Notably, immunosuppressant‐induced insulin resistance and hyperglycemia can exacerbate other complications. For instance, corticosteroid‐associated insulin resistance and resulting hyperinsulinemia increase the risk of posttransplant dyslipidemia by enhancing hepatic uptake of free fatty acids and cholesterol synthesis.^176^

In addition to the aforementioned insulin resistance, the mechanisms of immunosuppressant‐induced posttransplant dyslipidemia include abnormal cholesterol and lipoprotein metabolism.^177^, ^178^ First, corticosteroids and cyclosporine can increase the activity of key enzymes in cholesterol biosynthesis, such as 3‐hydroxy‐3‐methylglutaryl coenzyme A (HMG‐CoA) reductase, thereby enhancing the synthesis of very low‐density lipoprotein (VLDL) cholesterol.^177^ Second, cyclosporine can also impair the body's ability to remove low‐density lipoprotein (LDL) and VLDL by interfering with LDL receptor binding to LDL and decreasing lipoprotein lipase activity.^179^ Regarding posttransplant hypertension, immunosuppression, particularly CNI‐induced vasoconstriction and sodium retention, plays a crucial role. First, CNIs such as cyclosporine can lead to a reduction in vasodilators (e.g., nitric oxide) and an elevation in vasoconstrictors (e.g., endothelin and angiotensin II).^180^, ^181^ Second, CNIs may cause sodium retention by mediating aldosterone levels and sympathetic activation, which are thought to be strongly associated with hypertension.^182^, ^183^, ^184^ Moreover, the organ toxicity of immunosuppressants should not be disregarded. CNI‐related nephrotoxicity is believed to be closely associated with posttransplant chronic kidney disease, and the resulting endothelial dysfunction, alterations in vascular tone, and vascular calcification are all risk factors for posttransplant hypertension.^185^, ^186^, ^187^ It is noteworthy that the aforementioned metabolic complications, such as PTDM, posttransplant dyslipidemia, and hypertension, can act as risk factors that indirectly elevate the risk of posttransplant cardiovascular diseases.^51^

#### 3.3.2 The critical role of infection

In addition to the adverse effects of immunosuppressants, infection represents a significant factor contributing to metabolic disorders and cardiovascular diseases following transplantation. Viral infections, particularly CMV and HCV, play a crucial role in the development of metabolic complications. Currently, several retrospective cohort studies have identified CMV infection as a risk factor for PTDM.^188^, ^189^ Furthermore, numerous clinical studies have demonstrated that prior infection with hepatitis viruses, especially HCV, is associated with the incidence of PTDM.^189^, ^190^, ^191^ Notably, the study by Roccaro et al.^192^ confirmed an independent correlation between HCV eradication and a reduction in PTDM incidence following liver transplantation. Viral infection leading to pancreatic β‐cell damage is one of the mechanisms underlying PTDM. Substantial evidence supports the deleterious effects of CMV and HCV infection on pancreatic β‐cells.^193^, ^194^ Specifically, viral infection directly induces cell death, and the subsequent proinflammatory cytokine‐mediated immune response serves as the primary mechanism.^193^, ^194^, ^195^ Additionally, chronic infection results in decreased pancreatic β‐cell secretory function, which is a significant contributor to diabetes development.^196^ Viral infections such as CMV and HCV can also reduce insulin sensitivity in transplant recipients through various pathways. Insulin receptor substrate (IRS) is a key molecule in downstream insulin signaling. Both CMV and HCV core protein (HCVCP) can promote insulin resistance by downregulating IRS levels.^197^, ^198^ Specifically, CMV protein can cause sustained activation of mTOR complex 1 (mTORC1), which in turn leads to IRS degradation through phosphorylation.^198^ Regarding HCV, HCVCP can lead to the phosphorylation of Serine 312 of IRS‐1, thereby causing IRS‐1 degradation.^199^ Moreover, HCV infection can inhibit IRS‐1 function by activating the mTOR/S6K1 pathway and disrupt glucose metabolism by downregulating the glucose transporter GLUT4 and upregulating the gluconeogenic enzyme PCK2, thereby inducing insulin resistance.^200^ Furthermore, HCVCP can promote insulin resistance by inducing endoplasmic reticulum stress in the liver and hepatocytes.^201^ Regarding hyperlipidemia, mounting evidence suggests that HCV infection plays a crucial role. For instance, HCV core protein downregulates microsomal triglyceride transfer protein, an enzyme that mediates lipid translocation to the endoplasmic reticulum membrane, thereby reducing the assembly of VLDL.^202^

The development of posttransplant hypertension is influenced by CMV infection in transplant recipients.^203^ Cheng et al.^204^ proposed that CMV infection promotes inflammatory responses and activates the renin–angiotensin system, leading to elevated arterial blood pressure. Furthermore, CMV infection can induce endothelial dysfunction by reducing responses to bradykinin and glyceryl trinitrate. CMV can also directly infect endothelial cells, trigger inflammation, and promote thrombosis by enhancing the expression of von Willebrand factor, intercellular adhesion molecule‐1, and vascular cell adhesion molecule‐1, thereby further compromising endothelial cell function.^205^, ^206^, ^207^, ^208^ The primary types of infections that increase the postoperative cardiovascular risk in transplant recipients include HCV and CMV. The study by Maggi et al.^209^ demonstrated that liver transplant patients with previous or current HCV infection have a higher risk of cardiovascular disease. HCV infection can induce chronic inflammation, which is believed to be closely associated with endothelial dysfunction.^210^, ^211^ Moreover, direct infection of cardiovascular tissues by HCV may also play a significant role. For instance, numerous studies have isolated HCV RNA from carotid plaque tissue and blood–brain barrier endothelial cells of infected individuals.^212^, ^213^ Additionally, the aforementioned HCV‐related dyslipidemia is an important factor in cardiovascular diseases such as atherosclerosis.^214^ Conversely, CMV infection has been extensively documented as a significant risk factor for cardiovascular disease.^215^, ^216^ Vascular endothelial damage and dysfunction caused by CMV infection are considered the foundation for various cardiovascular diseases, including atherosclerosis and acute myocardial infarction.^217^ Furthermore, CMV infection may cause impaired arterial reactivity, tachycardia, and hypotension by inducing arterial smooth muscle dysfunction, which is thought to be related to sympathetic nerve activation.^218^ It is noteworthy that the exceptionally high risk of cardiovascular disease caused by simultaneous infection with multiple viruses warrants special attention. For example, Aguilera et al.^219^ conducted a retrospective analysis of liver transplant patients hospitalized for HCV cirrhosis and found that CMV reactivation was associated with an increased risk of cardiovascular events.

## 4 THERAPEUTIC INTERVENTIONS AND MANAGEMENT STRATEGIES

The prevention and management of posttransplantation complications require a multifaceted approach that integrates insights from the clinical features and molecular mechanisms discussed in the previous sections. This section explores various therapeutic interventions and strategies aimed at reducing the incidence and severity of posttransplant complications. By discussing the current state of immunosuppressive regimens, infection prophylaxis, metabolic management, and targeted therapies for malignancies, this section highlights the importance of personalized, evidence‐based approaches to optimize posttransplantation outcomes. Furthermore, this section identifies key areas for future research and emphasizes the need for ongoing efforts to develop novel therapeutic modalities and biomarkers for early detection and intervention.

### 4.1 Immunosuppressive therapies and novel cellular therapies

To prevent rejection or GVHD associated with transplantation and to induce tolerance, immunosuppressive therapies are now widely used in posttransplantation patients. The three‐signal model of T‐cell activation and proliferation provides a basis for understanding the molecular mechanisms of immunosuppression.^220^ In this model, signal 1 is the presentation of foreign antigen by APCs to T cells, activation of their TCR, and signaling through the transduction apparatus of the CD3 complex. Signal 2 is an antigen‐nonspecific costimulatory signal that results from the interaction of multiple ligand molecules on the surface of the APC with multiple receptors on the surface of the T cell, such as CD28 and CD154. Both signal 1 and signal 2 activate signal transduction pathways, including the calcium–calmodulin‐dependent phosphatase pathway, the mitogen‐activated protein kinase pathway, and the NF‐κB pathway. Signal 3 refers to the stimulation of the cell cycle by increased production of IL‐2 through the IL‐2 receptor. This process requires the mTOR enzyme to translate mRNA translation and cell proliferation.^221^, ^222^

Immunosuppressive therapy is primarily divided into induction therapy and maintenance therapy, based on the different treatment objectives.^223^ Induction therapy primarily aims to rapidly and potently suppress the body's immune response in the short term before and after transplantation surgery to prevent the occurrence of acute rejection. It mainly includes various antibodies. These antibodies have different mechanisms of action, depending on their target. For example, belatacept blocks the interaction of APCs with CD28 molecules on T lymphocytes, thereby blocking signal 2.^224^ IL‐2 receptor antagonists, such as basiliximab, inhibit lymphocyte activation and replication, thereby blocking signal 3.^225^ Moreover, polyclonal antibodies, such as antithymocyte globulin, can recognize multiple markers and exhibit a broad immunosuppressive profile.^224^ Maintenance therapy aims to control chronic graft rejection and maintain stable graft survival in the long term. Commonly used drugs include CNIs, antimetabolites, mTOR inhibitors, and glucocorticoids. CNIs, including cyclosporine A and tacrolimus, primarily exert their immunosuppressive effects by inhibiting calcineurin.^226^ mTOR inhibitors, such as sirolimus and everolimus, exert their immunosuppressive effects by inhibiting the proliferation and differentiation of T cells and B cells, as well as antibody production.^227^, ^228^ Antimetabolites, such as azathioprine and MMF, inhibit lymphocyte proliferation by inhibiting nucleic acid synthesis.^222^, ^224^ Glucocorticoids, such as methylprednisolone and prednisone, exert immunosuppressive and anti‐inflammatory effects.^229^ It is crucial to note that these immunosuppressive regimens are based on appropriate combinations of drugs with different mechanisms of action and need to be tailored to the individual patient. Currently, a commonly used regimen is based on triple drug therapy containing CNIs, corticosteroids, and antimetabolites.^223^

Considering the immunocompromise and toxicity inevitably associated with immunosuppressants, cellular therapy, particularly Treg‐based therapy, is of great importance in reducing the use of immunosuppressive therapy in patients. Currently, several clinical trials have demonstrated that Treg cell therapy has shown promising results in renal, hepatic, and other SOT patients.^230^, ^231^, ^232^, ^233^, ^234^ For example, the results of the study by Sawitzki et al.^232^ (The ONE Study) showed that it is feasible and safe for living kidney transplant recipients to receive Treg cell therapy with a relatively lower risk of infection. Additionally, a study by Todo et al.^234^ demonstrated that living liver transplant recipients who received Treg therapy to induce immune tolerance had 7 out of 10 patients maintaining immunosuppression‐free status for more than 6 years. The safety and efficacy of this therapy need to be further confirmed by additional studies.

### 4.2 Prevention and treatment of infections

The prevention of common infections in transplant recipients requires a multifaceted and comprehensive approach. On the one hand, comprehensive pretransplant screening of donors and recipients for infectious diseases is crucial. According to the recommendations in the American Society of Transplantation Clinical Practice Guidelines, transplant recipients should be screened preoperatively for various pathogens, including human immunodeficiency virus (HIV), tuberculosis, CMV, influenza, and pneumococcus. Moreover, in patients with active or recent peritonitis, it is recommended to delay transplantation to minimize the risk of infection.^235^ On the other hand, the guidelines also recommend immunization of transplant recipients well before transplantation, including pneumococcal, measles, mumps, rubella, diphtheria, tetanus, and pertussis vaccines.^235^ Furthermore, the resumption of vaccinations should be delayed after transplantation until immunosuppression is minimized and the vaccine's efficacy is serologically confirmed.^236^ Additionally, the importance of preoperative prophylactic medication against the risk of infection should be emphasized. For instance, in transplant recipients at high risk of CMV infection, preoperative oral ganciclovir is beneficial for graft survival.^237^ For donors, screening of donor organs for HBV, HCV, HIV, tuberculosis, schistosomiasis, and other infections should be emphasized to prevent the transmission of latent infections from the donor through infected organs.^238^, ^239^

When posttransplantation infection occurs, the timely selection of the appropriate treatment regimen and continuous monitoring are crucial. On the one hand, appropriately reducing the use of immunosuppressants is beneficial for the recovery of the body's ability to fight infection and improves therapeutic efficacy. For instance, in patients with EBV‐associated PTLD, reducing the use of immunosuppressants can lead to remission in 23−86% of patients.^240^ On the other hand, it is crucial to select the appropriate highly effective drugs for treatment based on the infecting pathogen. For viral infections, ganciclovir/valganciclovir for CMV, rituximab for EBV‐associated PTLD, and tenofovir or entecavir for HBV have shown promising results.^33^, ^34^, ^35^, ^36^, ^37^, ^38^, ^39^, ^40^, ^41^, ^42^, ^43^, ^44^, ^45^, ^46^, ^47^, ^48^, ^49^, ^50^, ^51^, ^52^, ^53^, ^54^, ^55^, ^56^, ^57^, ^58^, ^59^, ^60^, ^61^, ^62^, ^63^, ^64^, ^65^, ^66^, ^67^, ^68^, ^69^, ^70^, ^71^, ^72^, ^73^, ^74^, ^75^, ^76^, ^77^, ^78^, ^79^, ^80^, ^81^, ^82^, ^83^, ^84^, ^85^, ^86^, ^87^, ^88^, ^89^, ^90^, ^91^, ^92^, ^93^, ^94^, ^95^, ^96^, ^97^, ^98^, ^99^, ^100^, ^101^, ^102^, ^103^, ^104^, ^105^, ^106^, ^107^, ^108^, ^109^, ^110^, ^111^, ^112^, ^113^, ^114^, ^115^, ^116^, ^117^, ^118^, ^119^, ^120^, ^121^, ^122^, ^123^, ^124^, ^125^, ^126^, ^127^, ^128^, ^129^, ^130^, ^131^, ^132^, ^133^, ^134^, ^135^, ^136^, ^137^, ^138^, ^139^, ^140^, ^141^, ^142^, ^143^, ^144^, ^145^, ^146^, ^147^, ^148^, ^149^, ^150^, ^151^, ^152^, ^153^, ^154^, ^155^, ^156^, ^157^, ^158^, ^159^, ^160^, ^161^, ^162^, ^163^, ^164^, ^165^, ^166^, ^167^, ^168^, ^169^, ^170^, ^171^, ^172^, ^173^, ^174^, ^175^, ^176^, ^177^, ^178^, ^179^, ^180^, ^181^, ^182^, ^183^, ^184^, ^185^, ^186^, ^187^, ^188^, ^189^, ^190^, ^191^, ^192^, ^193^, ^194^, ^195^, ^196^, ^197^, ^198^, ^199^, ^200^, ^201^, ^202^, ^203^, ^204^, ^205^, ^206^, ^207^, ^208^, ^209^, ^210^, ^211^, ^212^, ^213^, ^214^, ^215^, ^216^, ^217^, ^218^, ^219^, ^220^, ^221^, ^222^, ^223^, ^224^, ^225^, ^226^, ^227^, ^228^, ^229^, ^230^, ^231^, ^232^, ^233^, ^234^, ^235^, ^236^, ^237^, ^238^, ^239^, ^240^, ^241^ ^‐^ ^242^ For HIV infection or bacterial infections, the appropriate antibiotics should be chosen to prevent or control the infection. Furthermore, active tuberculosis requires a 6‐month treatment regimen with isoniazid, rifampicin, pyrazinamide, and ethambutol, using all four drugs for the first 2 months and isoniazid and rifampicin for the remaining 4 months.^243^

### 4.3 Metabolic and cardiovascular risk management

The treatment of patients with PTDM should be chosen according to different stages. Intravenous insulin infusion is usually used in the early postoperative stage, followed by a transition to subcutaneous insulin injections. For long‐term glycemic management of PTDM, long‐term insulin use is also crucial.^7^ Additionally, common posttransplant oral hypoglycemic agents include metformin, sulfonylureas, and repaglinide.^244^, ^245^, ^246^ Among these, metformin has many advantages as a potential first‐line agent for posttransplant PTDM.^247^ Shivaswamy et al.^248^ demonstrated that in rats, metformin tended to reduce tacrolimus/sirolimus treatment‐induced endocrine cell apoptosis and ameliorate hyperglycemia induced by these immunosuppressive agents. Moreover, several studies have not observed any impairment or significant side effects of metformin on long‐term graft survival, suggesting its relative safety.^249^, ^250^ For patients with posttransplant dyslipidemia, statins are effective in lowering LDL levels and are recommended as the first‐line treatment and drug of choice for posttransplant dyslipidemia.^178^, ^179^, ^180^, ^181^, ^182^, ^183^, ^184^, ^185^, ^186^, ^187^, ^188^, ^189^, ^190^, ^191^, ^192^, ^193^, ^194^, ^195^, ^196^, ^197^, ^198^, ^199^, ^200^, ^201^, ^202^, ^203^, ^204^, ^205^, ^206^, ^207^, ^208^, ^209^, ^210^, ^211^, ^212^, ^213^, ^214^, ^215^, ^216^, ^217^, ^218^, ^219^, ^220^, ^221^, ^222^, ^223^, ^224^, ^225^, ^226^, ^227^, ^228^, ^229^, ^230^, ^231^, ^232^, ^233^, ^234^, ^235^, ^236^, ^237^, ^238^, ^239^, ^240^, ^241^, ^242^, ^243^, ^244^, ^245^, ^246^, ^247^, ^248^, ^249^, ^250^, ^251^ It is worth noting that statins are also believed to improve endothelial function, thereby benefiting cardiovascular health.^252^ Furthermore, drugs such as fibrates, niacin, and HMG‐CoA reductase inhibitors have shown promising therapeutic effects on posttransplant dyslipidemia due to their ability to LDL cholesterol levels.^178^, ^179^, ^180^, ^181^, ^182^, ^183^, ^184^, ^185^, ^186^, ^187^, ^188^, ^189^, ^190^, ^191^, ^192^, ^193^, ^194^, ^195^, ^196^, ^197^, ^198^, ^199^, ^200^, ^201^, ^202^, ^203^, ^204^, ^205^, ^206^, ^207^, ^208^, ^209^, ^210^, ^211^, ^212^, ^213^, ^214^, ^215^, ^216^, ^217^, ^218^, ^219^, ^220^, ^221^, ^222^, ^223^, ^224^, ^225^, ^226^, ^227^, ^228^, ^229^, ^230^, ^231^, ^232^, ^233^, ^234^, ^235^, ^236^, ^237^, ^238^, ^239^, ^240^, ^241^, ^242^, ^243^, ^244^, ^245^, ^246^, ^247^, ^248^, ^249^, ^250^, ^251^, ^252^, ^253^ Calcium channel blockers, which antagonize the vasoconstrictive effects of CNIs with fewer side effects, are considered first‐line agents in the management of posttransplant hypertension.^182^, ^183^, ^184^, ^185^, ^186^, ^187^, ^188^, ^189^, ^190^, ^191^, ^192^, ^193^, ^194^, ^195^, ^196^, ^197^, ^198^, ^199^, ^200^, ^201^, ^202^, ^203^, ^204^, ^205^, ^206^, ^207^, ^208^, ^209^, ^210^, ^211^, ^212^, ^213^, ^214^, ^215^, ^216^, ^217^, ^218^, ^219^, ^220^, ^221^, ^222^, ^223^, ^224^, ^225^, ^226^, ^227^, ^228^, ^229^, ^230^, ^231^, ^232^, ^233^, ^234^, ^235^, ^236^, ^237^, ^238^, ^239^, ^240^, ^241^, ^242^, ^243^, ^244^, ^245^, ^246^, ^247^, ^248^, ^249^, ^250^, ^251^, ^252^, ^253^, ^254^ Moreover, thiazide diuretics have demonstrated antihypertensive efficacy and safety in posttransplant hypertension.^255^ For posttransplant cardiovascular disease, it is crucial to manage the aforementioned metabolic complications.^56^ Notably, for metabolic complications and cardiovascular disease, lifestyle modifications such as smoking cessation and exercise are essential for prevention and improving prognosis.^256^, ^257^

For many transplant‐related metabolic and cardiovascular diseases, it is crucial to minimize the potential risk of complications associated with immunosuppressive therapy. In fact, several immunosuppressive agents have a relatively lower risk of complications and are considered more favorable options. A 12‐month, multicenter, prospective, randomized controlled trial by Wissing et al.^258^ found that therapeutic switching from tacrolimus to cyclosporine A significantly improved glucose metabolism in renal transplant recipients postoperatively and had the potential to reverse PTDM in the first year after transplantation. A randomized controlled trial by Kim et al.^259^ demonstrated that everolimus exhibited lower insulin resistance compared with low‐dose tacrolimus. Moreover, a study by Wen et al.^260^ demonstrated that the costimulation blocker belatacept, alone or in combination with tacrolimus, had a lower risk of PTDM compared with tacrolimus alone. However, the effect of tacrolimus on posttransplant hyperlipidemia was less pronounced than that of cyclosporine, and replacing cyclosporine with tacrolimus led to a 25% reduction in LDL cholesterol.^261^ Moreover, numerous studies have shown that the use of belatacept, either without or with reduced CNI exposure, can lower posttransplant lipid levels.^262^, ^263^ In patients with posttransplant hypertension, tapering, discontinuing, or avoiding corticosteroids and CNIs in favor of belatacept or mTOR inhibitors can be effective in controlling blood pressure.^262^, ^263^, ^264^ ^‐^ ^265^ Considering the significance of each metabolic complication in the development of cardiovascular disease, the impact of these immunosuppressive regimen changes on cardiovascular disease risk has also garnered considerable research attention. For instance, Vincenti et al.^266^ found a lower risk of serious cardiac events compared with cyclosporine by evaluating the long‐term safety profile of belatacept over a 5‐year period. Table 2 summarized the therapeutic interventions and management strategies in posttransplant complications.

**TABLE 2:** Summary of therapeutic interventions and management strategies in posttransplant complications.

Complications	Interventions	Details	References
Immune rejection and GVHD	Immunosuppressants	Calcineurin inhibitors, such as cyclosporine A and tacrolimus, inhibit calcineurin activity	226
		mTOR inhibitors, such as sirolimus and everolimus, inhibit the proliferation and differentiation of T and B cells	227, 228
		Antimetabolites, such as azathioprine and mycophenolate, inhibit nucleic acid synthesis	229, 230
		Glucocorticoids, such as methylprednisolone and prednisone, have immunosuppressive and anti‐inflammatory effects	231
	Novel cellular therapy	Treg cell therapy	230, 231, 232, 233, 234
Infection	Pathogen screening	HIV, tuberculosis, cytomegalovirus, and other pathogens	235, 238, 239
	Vaccination	Pneumococcal, measles, mumps, and rubella vaccines	236
	Antiviral drugs	Ganciclovir and so on	33, 241, 242
	Tuberculosis treatment scheme	Isoniazid, rifampicin, and so on	243
Metabolic and cardiovascular risk	Drug therapy	Insulin and oral hypoglycemics such as metformin are used to manage diabetes	244, 248, 249, 250
		Statins are used to lower LDL cholesterol and improve cardiovascular health	251, 252
		Bile acid sequestrants, niacin, and HMG‐CoA reductase inhibitors are used to reduce LDL cholesterol	254
		Calcium channel blockers are used to antagonize the vasoconstriction caused by CNIs	255
		Diuretics such as thiazides are used to treat posttransplant hypertension	257
	Conversion of immunosuppressive therapy	Converting from tacrolimus to cyclosporine, everolimus, or belatacept can reduce the risk of diabetes	258, 259, 260
		Using belatacept and other agents can relatively reduce the risk of dyslipidemia	262, 263
		Using belatacept and mTOR inhibitors can help control blood pressure	264, 265

Abbreviations: CNIs, calcineurin Inhibitors; GVHD, graft‐versus‐host disease; HIV, human immunodeficiency virus; HMG‐CoA, 3‐hydroxy‐3‐methylglutaryl‐coenzyme A; LDL, low‐density lipoprotein; mTOR, mammalian target of rapamycin.

### 4.4 Diagnosis and treatment of posttransplantation cancers

The high incidence and poor prognosis of posttransplant cancers underscore the importance of screening and early detection. Individualized cancer screening protocols for transplant recipients can help identify and intervene in cancers as early as possible, thereby improving prognosis. Considering that immune disorders are critical for posttransplantation tumorigenesis, the potential value of ILs as diagnostic markers for posttransplantation cancers has garnered considerable attention. Pontrelli et al.^267^ identified differentially expressed genes in patients with and without posttransplantation malignancies. The results showed that these differentially expressed genes were closely associated with the cancer pathway and that the IL‐27‐related genes were the most downregulated.^267^ Zhang et al.^268^ demonstrated that, compared with primary cutaneous squamous cell carcinoma (SCC), the number of IL‐22‐secreting CD8+ T cells in skin SCC tissues after skin transplantation was higher, and the expression of IL‐22 receptor in tissues was more diffuse. Furthermore, they found that increased IL‐22 and IL‐22R expression accelerated tumor growth in transplant patients.^268^ Moreover, T‐lymphocyte‐associated cell surface markers demonstrated promising predictive value. CD200, a membrane protein with immunosuppressive function expressed in hematopoietic tumors, suppresses the body's antitumor immunity by binding to the receptor CD200R, inducing Treg expression, and inhibiting tumor‐specific T‐cell function.^269^, ^270^, ^271^ The study by Vaughan et al.^272^ suggests the potential value of CD200 in posttransplantation cancers. Their study showed that 23.7% (nine out of 38) of PTLD patients exhibited CD200 positivity and that tumor cells exhibited membrane and cytoplasmic CD200 positivity. Furthermore, they found that PTLD patients with CD200 positivity had higher levels of Tregs compared with those with CD200 negativity.^272^ CD57 is a marker expressed on highly differentiated T cells, and an increased frequency of CD57‐positive T cells has been associated with various cancers. The reduced proliferative capacity of these cells suggests that the organism is in a state of chronic immune activation and senescence.^273^, ^274^ Bottomley et al.^275^ demonstrated that in kidney transplant recipients with cutaneous SCC, participants who highly expressed CD57 were more likely to develop SCC during follow‐up and tended to develop and experience recurrence earlier. They concluded that the percentage of CD8+ T cells expressing CD57 is a robust immune predictor of SCC occurrence and recurrence in long‐term, high‐risk kidney transplant recipients.^275^ Courivaud et al.^276^ found that CMV‐exposed renal transplant recipients were found to have a higher cumulative incidence of tumors compared with the unexposed population and a relatively shorter mean time to tumor onset. Notably, their results also demonstrated that CD57‐depleted T cells were significantly expanded in CMV‐exposed patients.^276^ Collectively, these studies suggest the potential significance of CD57 in predicting posttransplantation cancers. Moreover, the efficacy of EBV DNA for the diagnosis of PTLD has been widely recognized as a risk factor for the development of PTLD.^277^ A study by Rosselet et al.^278^ demonstrated that in seven out of 10 EBV‐positive PTLD patients, serum EBV DNA levels could be monitored in five of them. In contrast, EBV DNA was not detected in any of the 38 control subjects. Furthermore, a study by Suresh et al.^279^ demonstrated that in pediatric renal transplant recipients, an acute elevation of urinary CXCL10/creatinine ratio was associated with the development of transplant‐associated Burkitt's lymphoma, suggesting its potential value as a diagnostic biomarker.

**FIGURE 3:**
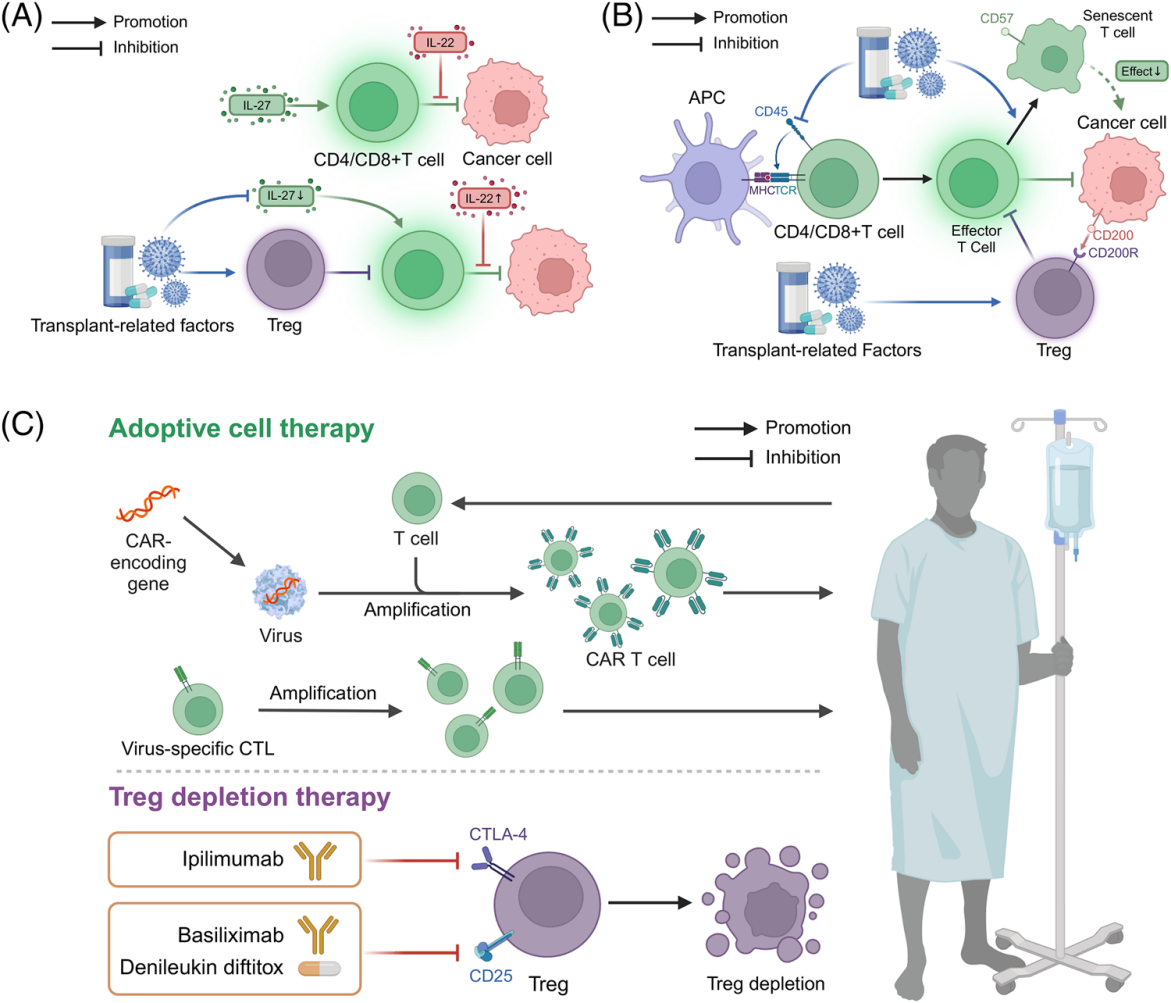
Diagnostic biomarkers and therapeutic strategies for posttransplant cancers. (A) Interleukins. IL‐27 can promote the anticancer response of T cells and has an inhibitory effect on Treg activity. IL‐22 released by T cells is considered to be associated with the occurrence and development of posttransplant cancers originating from epithelial tissues. Both of these cytokines have potential value as diagnostic biomarkers. (B) Cell surface biomarkers. CD45 on T cells can promote the activation of naïve T cells by APCs, but transplant‐related factors may inhibit CD45, affecting T‐cell function. CD200, expressed on the cell surface in some hematopoietic system cancers, can activate Tregs, thereby inhibiting the anticancer effects of effector T cells. CD57 is expressed on late‐stage T cells, which lack proliferative capacity, resulting in weakened anticancer effects. These markers have the potential to serve as diagnostic biomarkers. (C) Adoptive T‐cell therapy and Treg depletion therapy hold significant value for treating posttransplant cancers. ACT includes CAR‐T cell therapy and antiviral ACT. CAR‐T cell therapy involves the genetic modification of patient T cells by introducing CAR genes, allowing them to recognize cancer antigens with high specificity. The modified CAR‐T cells are expanded in vitro and then reinfused into the patient's body to enhance the immune system's attack on cancers. Antiviral ACT involves the in vitro expansion of posttransplant cancer‐specific T cells, which are then reinfused into the patient's body to rebuild virus‐specific immunity, thereby inhibiting cancer development. Treg depletion therapy primarily utilizes drugs targeting CTLA‐4, such as ipilimumab, drugs targeting CD25, such as basiliximab, and denileukin diftitox, which can inhibit the procancer effects of Tregs, thereby enhancing the anticancer effects of the immune system. This figure was created using tools provided by Biorender.com (accessed July 2, 2024).

Targeting the mechanisms of posttransplantation cancer development and progression is crucial for prevention and treatment. For example, sirolimus, which can reduce the activating effect of IL‐2 on T cells by inhibiting the mTOR receptor, has been used for immunosuppression in transplantation medicine, particularly after renal transplantation.^280^, ^281^ Notably, despite their immunosuppressive effects, some mTOR inhibitors can also suppress posttransplant tumorigenesis under certain circumstances.^282^ A randomized controlled trial by Knoll et al.^283^ demonstrated that a combination regimen was associated with a reduced risk of malignant tumors and nonmelanoma skin cancers in transplant recipients, compared with an immunosuppressive regimen without sirolimus. A meta‐analysis by Yanik et al.^284^ revealed that sirolimus use in renal transplant recipients was associated with a lower risk of renal carcinogenesis. Considering the central role of the PI3K/Akt/mTOR pathway in the growth, proliferation, and other functions of cancer cells, this may be a potential mechanism by which mTOR inhibitors suppress tumorigenesis after transplantation.^285^ Given the dual role of mTOR inhibitors in suppressing posttransplantation rejection and reducing the risk of posttransplantation tumorigenesis, they are essential for the prevention and treatment of specific types of posttransplantation cancers. Moreover, targeted therapies aimed at inhibiting Tregs in the management of posttransplantation tumors have garnered significant interest. Numerous studies have investigated the potential efficacy of ipilimumab (anti‐CTLA‐4), basiliximab (anti‐CD25), and denileukin diftitox in depleting intratumoral Tregs.^286^, ^287^, ^288^, ^289^ Similarly, targeting the chemokine receptor CCR8 to inhibit Tregs has gained traction in recent years, as it is believed to facilitate dose‐dependent, potent, and long‐lasting antitumor immune responses.^290^ Furthermore, targeted therapies directed against the transcription factor Foxp3, such as TCR mimetic antibodies that selectively bind to Foxp3‐expressing Tregs, have demonstrated considerable promise in Treg depletion.^291^ At present, these Treg depletion strategies have exhibited favorable antitumor effects in various malignancies, including gastric, head and neck, and lung cancers.^287^, ^288^, ^289^, ^290^, ^291^, ^292^, ^293^ In the context of treating posttransplantation tumor recurrence following hematologic transplantation, several Treg depletion approaches have yielded promising outcomes.^288^, ^289^, ^290^, ^291^, ^292^, ^293^, ^294^ For patients with recurrent multiple myeloma undergoing autologous stem cell transplantation (ASCT), two strategies—the elimination of Tregs in ASCT grafts using anti‐CD25 microbeads and the in vivo depletion of Tregs using basiliximab—significantly reduced the frequency of Tregs post‐ASCT.^288^ Among recipients of allo‐HSCT, the inhibition of Tregs using antitumor necrosis factor receptor type 2 (TNFR2) therapy was demonstrated to elicit an allogeneic response and consequently induce substantial antitumor effects.^294^ Moreover, for patients with recurrent hematologic malignancies following allo‐HSCT, ipilimumab has been shown to restore the body's antitumor responsiveness by enhancing the graft‐versus‐tumor effect.^295^ In summary, while additional studies and clinical validation are still required, the potential utility of Treg depletion strategies in managing posttransplantation tumors presents a promising therapeutic approach. The therapeutic potential of allogeneic cell therapy (ACT), including allogeneic T‐cell therapy and antiviral ACT, has garnered significant attention. In SOT, such as kidney and heart transplantation, numerous case reports have indicated that chimeric antigen receptor T‐cell (CAR‐T) therapy has demonstrated therapeutic efficacy in PTLD.^296^, ^297^, ^298^ Moreover, evidence suggests that CAR‐T therapy may also be effective in treating PTLD and drug‐resistant or refractory PTLD after allo‐HSCT.^299^, ^300^ Furthermore, a systematic evaluation study by Liu et al.^301^ demonstrated that, in addition to post‐HSCT PTLD, virus‐specific CTLs targeting EBV (EBV‐CTLs) exhibited safe therapeutic efficacy. Donor‐derived CMV‐specific CTLs (CMV‐CTLs) reduce the severity of CMV infection after transplantation, which may increase the risk of tumorigenesis in renal transplant recipients.^302^, ^303^ Moreover, Blyth et al.^304^ observed that donor‐derived CMV‐CTLs reduced the need for CMV‐directed drug therapy in allogeneic stem cell transplant recipients. Figure 3 summarized the diagnostic and therapeutic strategies for posttransplant cancers. Additionally, Table 3 compiles recent preclinical and clinical studies on the treatment of posttransplant complications from the past five years.

**TABLE 3:** Current summary of preclinical animal or cell‐based experiments and clinical research on the treatment of posttransplantation complications.

Complications	Therapeutic strategy	Study types	Functions of discovered strategy	References
GVHD	MSCs	In vitro, in vivo	Enhance thymic regeneration, modulating oxidative stress, reducing PD‐1 expression on T cells, and polarizing macrophages to an anti‐inflammatory state.	305, 306, 307
	Donor expanded Tregs	In vitro, in vivo	Enhances NK cell recovery and function, providing early immunity against tumors and pathogens post‐allo‐HSCT	308
	CB‐SCs	In vitro, in vivo	Modulate allogeneic CD4+ T cell activation and proliferation using soluble factors and extracellular vesicles	309
	JAK inhibitors	In vivo	Inhibit T cell activation, modulating Th cell differentiation, and disrupting the IFN‐g/CXCR3/CXCL10 axis	310
Rejection	MSCs	In vitro, in vivo	Downregulate TBK1/IRF3 phosphorylation and reduce IFN‐γ production (in combination with tacrolimus), and promote macrophage polarization to the M2 phenotype via CXCR4 regulation	311, 312
	IL‐10–BM‐MSCs	In vivo	Upregulate lncRNA 003946 in infiltrating CD68+ macrophages, thereby enhancing immunoinhibitory effects and reducing antigen presentation	313
	miRNAs	In vivo	Induce M2 polarization of macrophages through the PI3K/Akt (miR‐27a‐5p) and Myd88/TRAF6 pathways (miR‐505‐5p), thereby enhancing immunoinhibitory effects and reducing inflammation	314, 315
	MICs	In clinical research	Increase tolerogenic Bregs that produce IL‐10, leading to specific donor tolerance without adverse immune reactions	316
	IL‐35‐ASCs	In clinical research	Utilize IL‐35‐ASC‐derived exosomes to upregulate Tregs and enhance immunosuppressive properties, thereby prolonging graft survival	317
	Interleukin‐10	In vitro, in vivo	Downregulate miR‐155 and activates SOCS5, promoting macrophage M2 polarization and relieving chronic rejection after heart transplantation	318
	Interleukin‐37	In vitro, in vivo	Reduce Th1 and Th17 cells, increase Tregs, decrease TNF‐α and IFN‐γ levels, increase IL‐10 levels, and inhibit p‐mTOR expression in CD4+ cells	319
Malignancy	CAR‐T	In clinical research	CAR‐T therapy shows promising efficacy and safety in treating relapsed/refractory SOT‐related PTLD, with 22 patients included in the study, achieving a 64% overall response rate and approximately one‐third of patients achieving sustained remission	300
	Virus‐specific CTLs	In vitro	Immunosuppressive drug‐resistant HBV‐specific TCR‐redirected T cells can effectively lyse circulating HBV‐HCC cells in whole blood, even in the presence of immunosuppressants, potentially preventing posttransplant tumor recurrence	320
PTDM	SGLT2 inhibitors	In clinical research	Improve glycemic control, reduce body weight, blood pressure, proteinuria, and serum uric acid levels, while increasing serum magnesium and hemoglobin levels in PTDM	321, 322
	DPP4 inhibitors	In clinical research	Improve glycemic control and reduce body weight in PTDM patients by enhancing glucose excretion through the kidneys	323, 324
	FXR agonists	In vitro, in vivo	Inhibit gluconeogenesis and promoting glucose uptake through FXR activation in a PGC1α/FOXO1‐dependent manner	325
	GLP1R agonists	In clinical research	Improve glycemic control and reduce insulin requirements in PTDM patients without affecting immunosuppressant levels or kidney function	326, 327
	Sitagliptin	In clinical research	May help prevent the development of PTDM by improving glucose tolerance when administered early posttransplant	328
Dyslipidemia	PCSK9 inhibitors	In clinical research	Effectively and safely reduce lipid levels in heart transplant patients with hyperlipidemia unresponsive to statins, without adverse effects on liver and kidney parameters	329, 330
Cardiovascular diseases	RASBs	In clinical research	Potentially reduce stroke events in kidney transplant recipients by mitigating cardiovascular risks	331
	SGLT2 inhibitors	In clinical research	Reduce the risk of major adverse cardiovascular events, particularly myocardial infarction and cardiovascular mortality, in kidney transplant recipients with diabetes	332
	PCSK9 inhibitors	In vivo, in clinical research	Reduce vascular stenosis, inhibit inflammatory responses, and suppress vascular smooth muscle cell migration and proliferation through mechanisms involving the downregulation of NLRP3 inflammasome activation	333, 334

Abbreviations: allo‐HSCT, allogeneic hematopoietic stem cell transplantation; ASCs, adipose‐derived mesenchymal stem cells; Bregs, regulatory B cells; CAR‐T, chimeric antigen receptor T‐cell; CB‐SCs, cord blood derived‐multipotent stem cells; CTLs, cytotoxic T cells; CXCL, C‐X‐C motif chemokine ligand; CXCR, C‐X‐C motif chemokine receptor; DPP4, dipeptidyl peptidase 4; FXR, farnesoid X receptor; GLP1R, glucagon‐like peptide 1 receptor; GVHD, graft‐versus‐host disease; HBV, hepatitis B virus; HCC, hepatocellular carcinoma; IFN‐γ, interferon‐gamma; IL, interleukin; JAK, Janus kinase; MICs, modified immune cells; MSCs, mesenchymal stromal cells; mTOR, mammalian target of rapamycin; NLRP3, NOD‐, LRR‐, and pyrin domain‐containing protein 3; PCSK9, proprotein convertase subtilisin/kexin‐9; PD‐1, programmed cell death protein‐1; PTLD, posttransplant lymphoproliferative disorders; RASBs, renin–angiotensin system blockers; SGLT2, sodium–glucose cotransporter 2; SOT, solid organ transplantation; TCR, T cell receptor; TNF‐α, tumor necrosis factor‐alpha; Tregs, regulatory T cells.

### 4.5 Pharmacogenetics and immunogenetics in personalized immunosuppression and risk stratification

Pharmacogenetics and immunogenetics have emerged as critical areas of investigation in personalized medicine, particularly in the realm of immunosuppression management and risk stratification for transplant recipients. Currently, one of the most extensively studied pharmacogenetic markers is the cytochrome P450 3A5 (CYP3A5) polymorphism, which significantly influences the metabolism of tacrolimus, a widely used immunosuppressant in transplantation medicine. Individuals possessing a *CYP3A5* expression genotype require a longer duration to achieve target tacrolimus plasma concentrations compared with nonexpressers.^335^ Given the pivotal role of immunosuppressants in posttransplantation complications, numerous studies are presently exploring the impact of *CYP3A5* expression on the risk of such complications. Research conducted by Cheng et al.^336^ demonstrated that among renal transplant recipients receiving tacrolimus, patients with the CYP3A5*1*1 genotype necessitated higher drug doses to attain target plasma concentrations, potentially resulting in insufficient immunosuppression and consequently elevating the risk of acute rejection. Multiple studies have corroborated this increased rejection risk associated with the CYP3A5*1 allele.^337^, ^338^ Furthermore, among allo‐HSCT recipients receiving tacrolimus, those expressing *CYP3A5* exhibited a higher incidence of GVHD.^339^ These findings suggest that pretransplant administration of immunosuppressants based on genotypes of genes such as *CYP3A5* may facilitate the achievement of desired therapeutic concentrations, thereby optimizing transplant outcomes and mitigating adverse effects associated with immune overactivation. Regarding immunogenetic markers, HLA typing remains a crucial factor in organ transplantation. Matching donor and recipient HLA alleles can significantly reduce the risk of graft rejection and improve long‐term outcomes.^340^ The degree of HLA compatibility between donor and recipient is inversely correlated with the risk of rejection.^341^ Consequently, adjusting the dosage of recipient immunosuppressants according to the degree of HLA matching may be advantageous in preventing immunosuppression‐related complications, such as infections. It is noteworthy that a substantial body of research has demonstrated the critical importance of matching ABO blood group antigens and MHC class I polypeptide‐related sequence A (MICA) between donor and recipient in controlling rejection.^342^, ^343^ Moreover, variations in genes encoding cytokines and their receptors have been associated with distinct immune responses, which may inform risk stratification and guide tailored immunosuppressive strategies.^344^ For instance, the study by Dukat‐Mazurek et al.^345^ confirmed a potential correlation between acute GVHD following HSCT and IL‐6 genotype C/C homozygosity. Additionally, they identified a significant protective effect of the IL‐10 gene GCC/ATA haplotype on the development and severity of GVHD.^345^

## 5 FUTURE OUTLOOK AND SUMMARY

**FIGURE 4:**
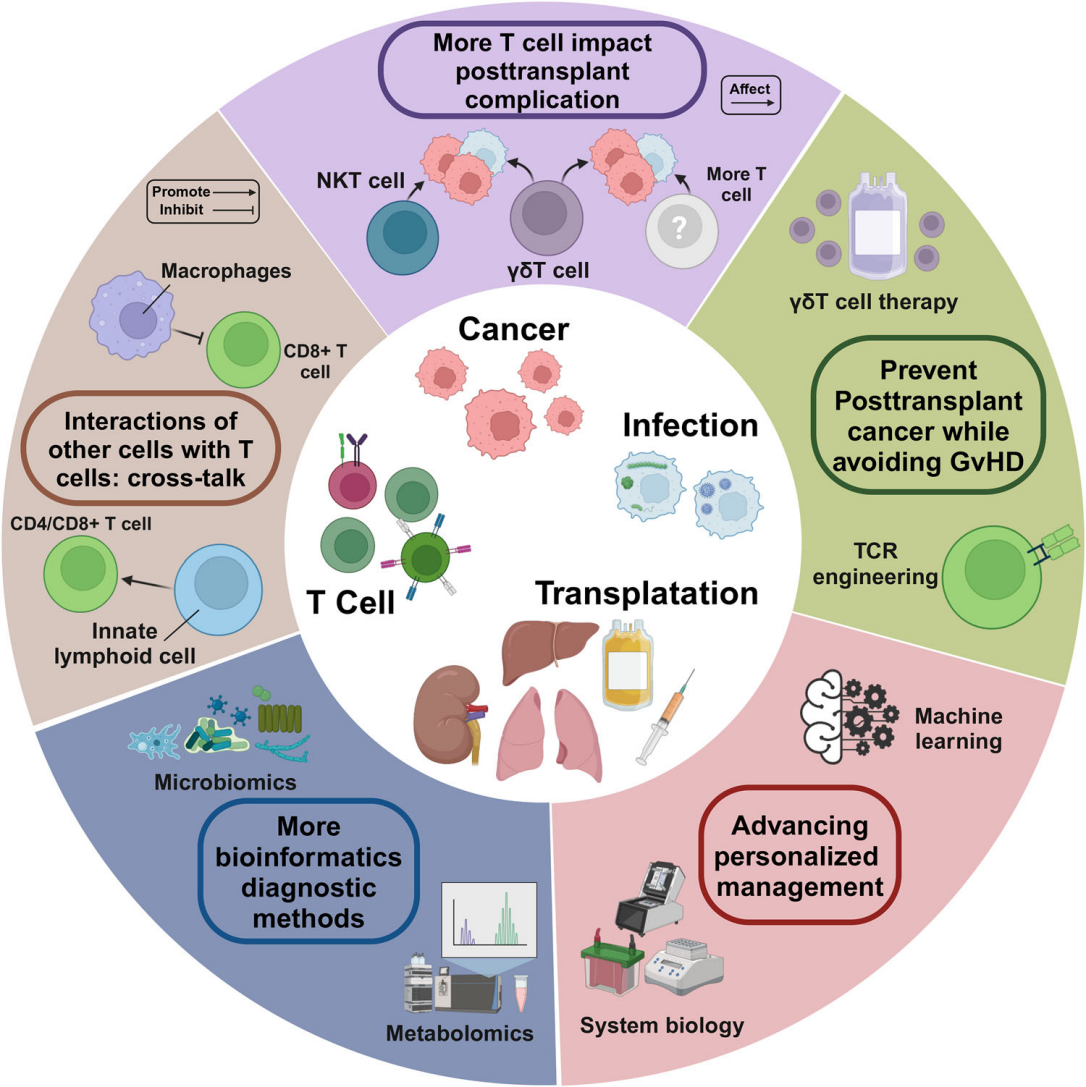
Future research directions regarding posttransplant complications. This figure was created using tools provided by Biorender.com (accessed July 24, 2024).

In summary, immune system dysfunction plays a crucial role in the development of posttransplantation complications. Despite extensive research efforts to elucidate the underlying mechanisms, several key aspects require further investigation. These include a more detailed understanding of the role of specific immune cell subsets in the pathogenesis of posttransplantation complications, the development of more accurate diagnostic methods for early detection and monitoring, and the identification of novel, effective prevention and treatment strategies. Here, we highlight four key points that warrant further exploration (Figure 4).

### 5.1 The role of more immune cells in posttransplant complications

In the previous section, we discussed the pivotal roles of CD4+ and CD8+ T cells in posttransplant complications. However, the involvement of additional T cell subsets in the development of posttransplant complications remains unclear. Indeed, accumulating evidence suggests that other T cell subsets can be equally influential in the development of posttransplant complications.^346^, ^347^ Natural killer T (NKT) cells are a distinct class of innate T lymphocytes that recognize lipid antigens presented by the MHC class I molecule CD1d. During liver transplantation‐associated infections with pathogens such as HBV or HCV, different NKT cell subtypes can eliminate infected cells and pathogens through direct cytotoxic effects and by promoting the activation of CTLs.^348^, ^349^ In antitumor immunity, NKT cells can sense tumor‐mediated changes in lipid metabolism by recognizing endogenous lipids and modulate anticancer immunity by secreting a series of cytokines.^346^ Indeed, numerous studies have observed an association between transplantation‐related factors and NKT cells.^350^, ^351^ Moiseev et al.^350^ demonstrated that higher levels of NKT cells in peripheral blood stem cell transplant donors were associated with impaired immune recovery in recipients treated with posttransplant cyclophosphamide. Furthermore, a study by Hodge et al.^351^ revealed that immunosuppressants, such as cyclosporine and prednisolone, inhibited the production of proinflammatory cytokines, including IFN‐γ and TNF‐α, by NKT cells. In addition to NKT cells, the potential role of gamma delta (γδ) T cells in posttransplantation complications is also of interest. In many cases, γδ T cells can directly recognize antigens through their γδ TCR without the involvement of APCs.^352^ In transplantation‐associated complications, γδ T cells can play multiple roles. On the one hand, certain subsets of γδ T cells may suppress the immune function of transplant recipients.^353^ In hematopoietic stem cell transplantation, IL‐17A‐producing γδ T cells can effectively enhance immunosuppression.^354^ In liver transplant recipients, γδ T cells have also been reported to be associated with hepatic allograft tolerance. Therefore, these γδ T cell‐associated immune impairments may increase the risk of posttransplant infections and tumorigenesis. On the other hand, in some cases, γδ T cells can control the reactivation of viruses, such as EBV and CMV, which may enhance their ability to achieve posttransplant tumor suppression.^355^ Currently, substantial evidence supports that transplantation‐related factors, such as the use of immunosuppressants and CMV infection, have a significant effect on γδ T cells.^347^, ^348^, ^349^, ^350^, ^351^, ^352^, ^353^, ^354^, ^355^, ^358^ Therefore, the impact of γδ T cells on posttransplantation cancers cannot be overlooked. However, there is still a paucity of studies to elucidate the precise role of T cell subsets, such as NKT and γδ T cells, in posttransplant infections or tumors. Therefore, more refined phenotyping criteria for T cell subsets need to be further investigated.

In addition, the crosstalk between other immune cells and T cells in the onset of impaired immune surveillance after transplantation warrants further investigation. In addition to NK cells and γδ T cells, other innate lymphoid cell (ILC) subpopulations, such as group 2 ILCs (ILC2s) and group 3 ILCs (ILC3s), play an equally important role in posttransplantation immunity.^357^ ILC2s can promote antigen‐specific CD4+ T cell responses through the expression of MHC class II molecules and enhance NK cell cytotoxicity and T cell responses by secreting IL‐13.^358^, ^359^ On the other hand, ILC3s promote CD4+ and CD8+ T cell infiltration in tumors by producing CXCL10.^360^ Furthermore, tumor‐associated macrophages (TAMs) also influence the antitumor immune function of T cells. Under specific circumstances, TAMs may inhibit CD8+ T cell recruitment by producing IL‐15/IL‐15 receptor alpha complexes while promoting Tregs by producing C‐C motif chemokine ligand 22.^361^, ^362^ Currently, the impact of these cells on posttransplantation infections or tumorigenesis needs to be further elucidated. Furthermore, it is necessary to thoroughly investigate whether other cells can also influence T cells, leading to impaired immune surveillance after transplantation.

### 5.2 Emerging clinical diagnostic technologies

The potential application of additional bioinformatics techniques in the diagnosis of posttransplantation complications requires further investigation. Given the central role of T cells in transplantation‐associated immunity, conventional techniques capable of identifying T cell‐associated biomarkers, such as single‐cell RNA sequencing (scRNA‐seq), spatial transcriptomics, and mass cytometry by time‐of‐flight, have been extensively employed in the diagnosis of posttransplantation complications, such as cancers.^363^, ^364^, ^365^ Recently, novel techniques have been employed to evaluate T cell function, such as TCR sequencing (TCR‐seq) and peptide–MHC multimer technology.^366^, ^367^, ^368^ These techniques have been applied to monitor posttransplant complications, such as the risk of immune rejection and T cell‐mediated antitumor immune responses. However, their potential application in the diagnosis of other posttransplant complications requires urgent investigation.^369^, ^370^, ^371^ Importantly, diagnosing posttransplantation complications from a multiomics perspective is a promising research avenue. The development of multiomics‐based biomarkers for posttransplant rejection is currently a highly active area of research.^372^ For instance, Lin and colleagues^187^ developed a novel bioinformatics approach for identifying microRNA markers closely associated with kidney transplant rejection by integrating multiomics data to construct a molecular interactome network. Moreover, genomics, transcriptomics, and proteomics related to T cells have been extensively employed in detecting posttransplantation cancers.^373^, ^374^, ^375^, ^376^ Furthermore, other omics approaches have gained increasing attention, particularly metabolomics, which has potential diagnostic value for posttransplantation complications due to its ability to monitor T cell function.^377^ For example, measuring the levels of mitochondrial and lipid metabolism in Tregs can reflect anti‐infective or antitumor immunity.^378^ Additionally, given the role of various viruses and bacteria in posttransplant infections and tumors, the value of microbiome analysis in this context has also garnered attention. For instance, a study by Tellez et al.^379^ demonstrated the reliability of microbial assay arrays in monitoring EBV infection in lymphoma. Although numerous studies suggest the potential value of multiomics approaches in diagnosing posttransplantation cancers, their application requires further investigation.

Interestingly, certain clinical diagnostic techniques focusing on T cells have potential applications in diagnosing posttransplant complications. Imaging is one such technique with potential applications in this context.^10^ For example, a study by Anthony et al.^380^ demonstrated that by injecting D‐luciferin into mice after bone marrow transplantation and using bioluminescence imaging, researchers could track specific T cell subpopulations and hypothesize about their contribution to GVHD at different time points. Notably, this noninvasive technique for monitoring T cell activity may also show promise in posttransplant tumor surveillance.^380^ Similarly, pathological analyses targeting T cells also have potential applications in diagnosing posttransplantation tumors. In patients with posttransplantation tumors, Datta et al.^1^ adequately assessed CD8+ T cell abundance in the tumor microenvironment by performing digital image analysis of tumor and adjacent tissue sections, finding that reduced abundance correlated with impaired cancer immune surveillance. Furthermore, Mohri et al.^381^ used microscopy to observe the presence of numerous large atypical cells in the background of dense T cell aggregates, using this pathological feature as evidence to support the diagnosis of classical Hodgkin lymphoma‐type posttransplant lymphoproliferative disorder following renal transplantation. In the future, it will be essential to investigate additional clinical diagnostic techniques focusing on T cells and explore their potential applications in diagnosing posttransplantation tumors.

### 5.3 Advancing personalized management with cutting‐edge technologies

Multiomics technologies have demonstrated exceptional performance in the accurate and comprehensive prediction of posttransplant complications, which provide crucial guidance for improving transplant patient outcomes. In the field of genomics, Ma et al.^382^ developed a 355‐gene transplant incidence panel encompassing cardiovascular disease, immunodeficiency, malignancy, and thrombosis. Their evaluation of this genetic panel, using exome sequencing data from 1590 kidney transplant recipients, revealed that approximately 9% of patients harbored variants predisposing them to these complications. This finding underscores the potential value of genetic testing in informing clinical decision‐making, such as implementing more intensive monitoring, adjusting immunosuppressive regimens, or initiating specialist referrals.^382^ Proteomics has garnered significant attention in posttransplantation complication research. Hu et al.^383^ conducted antibody array analyses on urine samples from kidney transplant recipients, identifying significantly elevated levels of 11 cytokines/chemokines in patients experiencing acute rejection compared with healthy controls. Their study further demonstrated that a combination of markers, including 10 kDa induced protein, IFN‐γ‐induced monokine and macrophage inflammatory protein‐1Delta, could differentiate between acute and chronic injury.^383^ Additionally, the application of other multiomics approaches, such as transcriptomics and metabolomics, in posttransplantation complication studies has gained increasing traction.^384^, ^385^ Systems biology adopts a holistic perspective, integrating multiomics data to generate and validate novel hypotheses regarding organ function and failure. This approach necessitates addressing data variability, noise, and scale, while combining experimental big data with computational models to reconstruct and elucidate biological processes.^386^ Sui et al.’s^387^ research exemplifies the potential of systems biology in posttransplantation complications. By integrating proteomics and genomics technologies, they explored the expression levels of transcription factors, microRNAs, and long noncoding RNAs in biopsy samples from kidney transplant patients with acute rejection and healthy controls, identifying characteristic markers through association analysis.^387^ Future research should focus on optimizing systems biology methods and integrating more multiomics data to develop accurate predictive models. These advancements aim to enable early detection and personalized treatment of posttransplantation complications, ultimately improving long‐term patient outcomes. Machine learning (ML) further enhances personalized management by leveraging large‐scale clinical and molecular data to identify patterns and predict outcomes with high accuracy. ML has been widely applied in D–R matching prediction prior to transplantation.^388^, ^389^ For instance, Briceño et al.^390^ employed artificial neural networks for D–R matching in liver transplantation, demonstrating superior performance in predicting graft survival and probability of graft loss compared with validated graft survival scores.^391^ ML‐based analyses hold promise for achieving precise D–R matching, potentially preventing posttransplantation complications. ML has also shown efficacy in predicting posttransplantation patient prognosis and exhibits significant potential in forecasting the risk of complications such as rejection, GVHD, and malignancy.^392^, ^393^, ^394^, ^395^, ^396^, ^397^ In many instances, ML demonstrates superior predictive capabilities compared with traditional methods. For example, Zare et al.^398^ reported that ML models outperformed conventional logistic regression in predicting acute rejection in liver transplant patients. Notably, ML techniques offer substantial advantages in processing unstructured data, such as medical imaging.^399^ In conclusion, advanced research methodologies including multiomics, systems biology, and ML are transforming the landscape of transplant medicine. These approaches are paving the way for more effective and personalized therapeutic interventions. The integrated application of these technologies is expected to significantly enhance the prevention, diagnosis, and treatment of posttransplantation complications, ultimately improving patients’ long‐term quality of life.

### 5.4 Balance of GVHD and posttransplantation cancers

Finally, the question of how to combat posttransplant tumors without causing GVHD is also an urgent one. Although the immunosuppressive effects of mTOR inhibitors are thought to counteract their anticancer effects, a substantial body of research suggests that mTOR inhibitors have a facilitating effect on the anticancer function of CD8+ T cells.^400^, ^401^ A randomized controlled trial by Knoll et al.^283^ demonstrated that, compared with an immunosuppressive regimen without sirolimus, the combination regimen was associated with a reduced risk of malignant neoplasms and nonmelanoma skin cancers in transplant recipients. A meta‐analysis by Yanik et al.^284^ revealed that sirolimus use in renal transplant recipients was associated with a lower risk of kidney cancer. This paradox arises from the fact that mTOR inhibitors are immunomodulators with a complex mechanism of action in adaptive immunity, capable of both promoting and suppressing immune responses.^400^ Therefore, the effect of mTOR inhibitors on CD8+ T cells may depend on the relative predominance of their immunostimulatory and immunosuppressive effects. Given the dual effects of mTOR inhibitors in suppressing posttransplantation rejection and reducing the risk of posttransplantation tumorigenesis, their potential application in preventing and treating specific types of posttransplantation tumors warrants further investigation. Furthermore, γδ T cells may offer a promising strategy for resolving this issue. Numerous studies have demonstrated that γδ T cells, which lack allogeneic heterozygosity, can be safely used in hematopoietic stem cell transplantation and can mediate natural antitumor responses.^402^, ^403^ Consequently, γδ T cells have potential applications for simultaneously preventing GvHD and posttransplantation tumors in specific types of transplantation. Numerous studies support this concept. For instance, in allo‐HSCT recipients, γδ T cells are not believed to cause GvHD and may promote graft‐versus‐leukemia effects.^404^ Furthermore, Wu et al.^405^, ^406^ observed that the proportion of CD27+Vδ1 Tregs, a subpopulation of γδ Tregs, in grafts transplanted with peripheral blood stem cells was negatively correlated with acute GVHD. They also found that γδ Tregs exhibited a cytotoxic effect on tumor cells following granulocyte colony‐stimulating factor treatment.^405^, ^406^ However, the specific mechanism of action and the potential role of γδ T cells in a broader range of transplantation settings require further investigation. Moreover, in addition to CAR‐T therapy, TCR engineering, a relay cell therapy, has also shown potential for suppressing tumors after transplantation. Currently, T cells recognizing EBV antigens through natural TCRs have been successfully used to treat EBV‐associated PTLD.^407^ Furthermore, modified TCR‐genetically engineered virus‐specific T cells targeting CMV and EBV were found to possess both antiviral activity and transgenic TCR‐mediated tumor‐targeting activity.^408^ Therefore, TCR engineering may also be a readily available cell therapy capable of specifically targeting tumors, and its further applications warrant additional investigation.

## 6 CONCLUSION

Posttransplant complications continue to pose significant challenges, limiting the long‐term survival and quality of life of transplant recipients. In this comprehensive review, we summarize the clinical features, risk factors, and molecular mechanisms underlying major posttransplant complications, including immune‐mediated, infectious, metabolic, and malignant complications.

The development of posttransplantation complications is driven by a complex interplay between immune activation, metabolic dysregulation, and immunosuppression. Innate and adaptive immune responses triggered by allogeneic antigens play a pivotal role in mediating graft rejection and GVHD. Metabolic complications, such as PTDM, dyslipidemia, and hypertension, are closely linked to the use of immunosuppressive agents and contribute substantially to cardiovascular morbidity in transplant recipients. Furthermore, posttransplant immunosuppression impairs the body's anti‐infective and antitumor immune responses, thereby increasing the patient's susceptibility to infections and malignancies. The reactivation of latent oncogenic viruses under immunosuppression is a key driver of posttransplant malignancy, underscoring the intricate relationship between immunosuppression and tumorigenesis. Drawing upon these mechanistic insights, we discuss potential prevention and intervention strategies for posttransplant complications. Optimizing immunosuppressive regimens to achieve a delicate balance between preventing rejection and minimizing adverse effects is of paramount importance. The development of novel immunosuppressive agents with higher specificity and lower toxicity holds promise for mitigating metabolic and cardiovascular complications. Moreover, enhanced infection prevention through vaccination, thorough D–R screening, and judicious use of antimicrobials can significantly reduce the burden of infectious complications. In the context of posttransplant malignancies, the implementation of antiviral medications, selection of appropriate immunosuppressive agents, and treatment with cellular therapies may improve the management of virus‐associated malignancies.

Despite significant advances in elucidating the mechanisms underlying posttransplant complications, several aspects remain to be fully understood. Future research should focus on elucidating the intricate interplay between immune, metabolic, and oncogenic pathways underlying these complications at the molecular level. Integrating multiomics approaches with systems biology will provide a more comprehensive understanding of the pathogenesis and facilitate the identification of novel therapeutic targets. Moreover, the development of personalized risk stratification and prevention strategies based on individual genetic and immunological profiles holds great promise for improving transplantation outcomes. A more profound understanding of the molecular mechanisms underlying these complications is crucial for developing effective prevention and treatment strategies. By integrating mechanistic insights with precision medicine approaches, we can optimize immunosuppression regimens, enhance complication monitoring, and implement targeted interventions to improve long‐term survival and quality of life for transplant recipients. Continuous research efforts in this field will usher in a new era of personalized transplant medicine, ultimately benefiting patients worldwide.

## AUTHOR CONTRIBUTIONS

Yongguang Liu, Ming Zhao, and Peng Luo provided direction and guidance throughout the preparation of this manuscript. Xiaoyou Liu, Junyi Shen, and Hongyan Yan wrote and edited the manuscript. Yongguang Liu, Ming Zhao, and Peng Luo reviewed and made significant revisions to the manuscript. Xiaoyou Liu, Junyi Shen, and Hongyan Yan revised and edited the manuscript. Jianmin Hu, Guorong Liao, Ding Liu, Song Zhou, Jie Zhang, Jun Liao, Zefeng Guo, Yuzhu Li, Siqiang Yang, Shichao Li, Hua Chen, Ying Guo, Min Li, Lipei Fan, and Liuyang Li collected and prepared the related papers. All authors read and approved the final manuscript.

## CONFLICT OF INTEREST STATEMENT

The authors declare that they have no conflict of interests.

## ETHICS STATEMENT

Not applicable.

## Data Availability

Not applicable.
